# Connectome analysis with diffusion MRI in idiopathic Parkinson's disease: Evaluation using multi-shell, multi-tissue, constrained spherical deconvolution

**DOI:** 10.1016/j.nicl.2017.11.007

**Published:** 2017-11-10

**Authors:** Koji Kamagata, Andrew Zalesky, Taku Hatano, Maria Angelique Di Biase, Omar El Samad, Shinji Saiki, Keigo Shimoji, Kanako K. Kumamaru, Kouhei Kamiya, Masaaki Hori, Nobutaka Hattori, Shigeki Aoki, Christos Pantelis

**Affiliations:** a Department of Radiology, Juntendo University Graduate School of Medicine, Tokyo, Japan; b Melbourne Neuropsychiatry Centre, Department of Psychiatry, The University of Melbourne & Melbourne Health, Parkville, VIC, Australia; c Melbourne School of Engineering, University of Melbourne, Melbourne, Australia; d Department of Neurology, Juntendo University Graduate School of Medicine, Tokyo, Japan; e Department of Computing and Information Systems, University of Melbourne, Parkville, Australia; f Department of Diagnostic Radiology, Tokyo Metropolitan Geriatric Hospital, Tokyo, Japan; g Department of Radiology, The University of Tokyo, Bunkyo, Tokyo, Japan; h Centre for Neural Engineering, Department of Electrical and Electronic Engineering, The University of Melbourne, Carlton, VIC, Australia

**Keywords:** CSD, constrained spherical deconvolution, CSF, cerebrospinal fluid, DW-MRI, diffusion-weighted magnetic resonance imaging, fODF, fiber orientation distribution function, GM, gray matter, MSMT-CSD, multi-shell, multi-tissue CSD, PD, Parkinson's disease, SVM, support vector machine, UPDRS, Unified Idiopathic Parkinson's Disease Rating Scale, WM, white matter, Connectome, Diffusion MRI, Diffusion tensor imaging, Lewy bodies, Neurodegenerative disorders, Support vector machine

## Abstract

Parkinson's disease (PD) is a progressive neurodegenerative disorder that affects extensive regions of the central nervous system. In this work, we evaluated the structural connectome of patients with PD, as mapped by diffusion-weighted MRI tractography and a multi-shell, multi-tissue (MSMT) constrained spherical deconvolution (CSD) method to increase the precision of tractography at tissue interfaces. The connectome was mapped with probabilistic MSMT-CSD in 21 patients with PD and in 21 age- and gender-matched controls. Mapping was also performed by deterministic single-shell, single tissue (SSST)-CSD tracking and probabilistic SSST-CSD tracking for comparison. A support vector machine was trained to predict diagnosis based on a linear combination of graph metrics. We showed that probabilistic MSMT-CSD could detect significantly reduced global strength, efficiency, clustering, and small-worldness, and increased global path length in patients with PD relative to healthy controls; by contrast, probabilistic SSST-CSD only detected the difference in global strength and small-worldness. In patients with PD, probabilistic MSMT-CSD also detected a significant reduction in local efficiency and detected clustering in the motor, frontal temporoparietal associative, limbic, basal ganglia, and thalamic areas. The network-based statistic identified a subnetwork of reduced connectivity by MSMT-CSD and probabilistic SSST-CSD in patients with PD, involving key components of the cortico–basal ganglia–thalamocortical network. Finally, probabilistic MSMT-CSD had superior diagnostic accuracy compared with conventional probabilistic SSST-CSD and deterministic SSST-CSD tracking. In conclusion, probabilistic MSMT-CSD detected a greater extent of connectome pathology in patients with PD, including those with cortico–basal ganglia–thalamocortical network disruptions. Connectome analysis based on probabilistic MSMT-CSD may be useful when evaluating the extent of white matter connectivity disruptions in PD.

## Highlights

• Connectomes mapped in Parkinson's disease (PD) using multi-shell tractography.
• Multi-shell tractography provided improved sensitivity to connectome pathology.
• Machine learning accurately predicted PD diagnosis based on connectome.
• Connectome pathology in PD was localized to basal ganglia-thalamocortical circuits.

## 1 Introduction

Parkinson's disease (PD) is the second most common neurological disorder after Alzheimer's disease, affecting 6 million individuals worldwide (Vos et al., 2016). The disease is characterized by motor symptoms (i.e., akinesia or bradykinesia, rigidity, and tremor) as well as non-motor symptoms such as cognitive impairment and psychiatric symptoms (Kalia and Lang, 2015). Motor symptoms primarily result from dopamine neurodegeneration in the substantia nigra pars compacta and striatum (putamen and caudate nucleus), which are the main targets of nigral dopaminergic axons. However, dopamine loss is associated with neuronal activity changes in cortico–basal ganglia–thalamocortical circuits, which are also linked to motor and non-motor symptoms (Rodriguez-Oroz et al., 2009). These circuits are large, functional, parallel, and segregated cortical–subcortical re-entrant circuits that are topographically and functionally divided into motor, associative, and limbic sub-circuits (Alexander et al., 1990). Therefore, disruption to these complex circuits may account for the many motor and non-motor symptoms observed in PD (Galvan et al., 2015).

Evidence for brain circuit and axonal pathology has been widely reported for PD. The pathological hallmark of PD is the presence of Lewy bodies and Lewy neurites, which are protein inclusions whose main constituent is fibrillary-aggregated α-synuclein (Spillantini et al., 1998). These abnormal aggregates predominantly occur at presynaptic sites, where they cause synaptic and axonal degeneration that can potentially result in widespread macroscopic disruption in connectivity.

The connectome is a network representation of whole-brain connectivity, which can be mapped to reveal circuit-based alterations in neurological and psychiatric conditions (Fornito et al., 2015, Fornito et al., 2012, Zalesky et al., 2016). Macroscale connectome studies combine diffusion-weighted magnetic resonance imaging (DW-MRI) and graph theory to generate a network representation of the brain, comprising gray matter (GM) brain regions (nodes) and axonal connections (edges) (Craddock et al., 2013).

Recent studies have used DW-MRI data to infer interregional structural connectivity (Behrens and Sporns, 2012) when evaluating connectome dysfunction in PD (Aarabi et al., 2015, Barbagallo et al., 2017, Galantucci et al., 2017, Li et al., 2016, Nigro et al., 2016, Shah et al., 2017, Tinaz et al., 2017). These studies report reduced structural connectivity between the basal ganglia, thalamic, limbic, frontal, temporal, and parietal regions in PD (Aarabi et al., 2015, Barbagallo et al., 2017, Galantucci et al., 2017, Kim and Park, 2016, Li et al., 2016, Nigro et al., 2016). Disrupted connectivity has shown associations with clinical and non-motor symptoms of PD (Barbagallo et al., 2017, Galantucci et al., 2017, Shah et al., 2017). Graph theory analyses have identified reduced global clustering and efficiency, as well as increased global path length (Nigro et al., 2016). In addition, local network measures have been shown to be disrupted in PD, including decreased “functional segregation,” as indicated by decreased local efficiency and clustering, and decreased “functional importance,” as indicated by decreased nodal strength and betweenness centrality in the basal ganglia, thalamus, limbic, frontal, temporal, parietal, and occipital cortices (Li et al., 2016, Nigro et al., 2016). Together, diffusion tensor imaging-based connectome studies have identified global and local connectome disruptions in PD in vivo.

These previous studies employed deterministic or probabilistic tractography to trace the trajectories of white matter (WM) streamlines (edges) and map structural connectomes per individual. A major limitation of deterministic fiber tracking based on unimodal diffusion tensor imaging is the difficulty in estimating neural fiber connections in a voxel in which there are crossing or kissing fibers (Mori and van Zijl, 2002). Probabilistic tractography algorithms that estimate multiple fiber directions have been proposed to overcome this limitation (Behrens et al., 2007), and one of the most commonly applied techniques is constrained spherical deconvolution (CSD), which can improve the reliability of whole-brain tractography by using a high-quality fiber orientation distribution function (fODF) (Jeurissen et al., 2011). However, CSD cannot yield accurate fODFs in voxels containing GM and cerebrospinal fluid (CSF) (Roine et al., 2014), and the method particularly struggles to reconstruct the GM–WM interface. Given that the connectome contains nodes (corresponding to GM regions) and edges between nodes (i.e., tractographic streamlines), accurate delineation of GM and WM is very important for accurate tractogram generation to be achieved.

To circumvent the limitations of deterministic tractography and the difficulties in resolving the GM–WM interface with existing CSD methods, we employed a new, multi-shell, multi-tissue CSD (MSMT-CSD) method (Jeurissen et al., 2014) to map whole-brain connectomes. To estimate the ODF in multiple tissues, MSMT-CSD employs multi-shell DW-MRI data with unique b-value dependences for different macroscopic tissue types (WM, GM, or CSF) (Jeurissen et al., 2014). Because MSMT-CSD can produce reliable volume fraction maps of WM, GM, and CSF directly from DW-MRI data, it can substantially increase the precision of the fODF and the resulting tractograms at tissue interfaces (Jeurissen et al., 2014).

We hypothesized that connectome disruption would be evident in PD, and that it would involve cortico–basal ganglia–thalamocortical networks (Rodriguez-Oroz et al., 2009). We further hypothesized that connectomes derived from the MSMT-CSD method would be more sensitive to connectivity deficits compared with conventional methods. To test this hypothesis, we compared connectivity strength as well as global and local graph theory metrics obtained by probabilistic MSMT-CSD with those obtained by deterministic single-shell, single tissue (SSST)-CSD tracking and probabilistic SSST-CSD tracking. Then, we used machine learning to evaluate which of MSMT-CSD, deterministic SSST-CSD tracking, and probabilistic SSST-CSD tracking yielded the highest accuracy for classifying cases and controls based on topological descriptors of the brain network.

## 2 Material and methods

### 2.1 Participants

This study was approved by the institutional review board of Juntendo University Hospital, Japan, and was conducted in accordance with the criteria of the Helsinki Declaration. Written informed consent was obtained from all participants before evaluation. In total, 21 patients with PD were recruited and the diagnosis of a movement disorder was confirmed by specialists according to the United Kingdom Parkinson's Disease Society Brain Bank criteria (Hughes et al., 1992). Disease severity was evaluated using the motor scores of the Unified Idiopathic Parkinson's Disease Rating Scale (UPDRS)-III (Martinez-Martin et al., 1994), and the Hoehn and Yahr staging scale (Hoehn and Yahr, 1967). All patients were receiving levodopa in combination with a dopamine decarboxylase inhibitor (benserazide or carbidopa) at the time of scanning, were required to have responded to antiparkinsonian therapy, and were required to have remained free of atypical parkinsonism at 12 months (or longer) after diagnosis. For comparison, we recruited 21 age- and gender-matched healthy controls with no history of neurological disease. The demographic and clinical characteristics of all participants are shown in Table 1.

**Table 1:** Demographic characteristics of the participants.

	Controls (n = 21)	Patients with PD (n = 21)	*χ*^2^/*t*	*P* value
Sex, male:female	8:13	12:9	1.53	0.35
Age in years, mean (SD)	63.7 (9.8)	64.5 (9.1)	− 0.27	0.78
Disease duration in years, mean (SD)	0	5.0 (3.0)	–	–
Hoehn–Yahr stage (SD)	0	1.5 (0.7)	–	–
UPDRS-III motor subscale score, median (SD)	0	14.4 (8.9)	–	–

Abbreviations NA, not applicable; NS, not significant; PD, Parkinson disease; UPDRS, Unified Idiopathic Parkinson's Disease Rating Scale; SD, standard deviation.

### 2.2 Image acquisition

Neuroimaging data were obtained on a 3.0-T system (Achieva; Philips Healthcare, Best, the Netherlands) equipped with an eight-channel head coil for sensitivity-encoding parallel imaging. DW-MRI was acquired at b-values of 1000 and 2000 s/mm^2^ along 32 uniformly distributed directions with a spin-echo echo-planar imaging scheme in an anterior–posterior phase encoding direction. The same diffusion directions were used for each shell. Each DW-MRI acquisition was complemented with a non-weighted diffusion image (b = 0 s/mm^2^). Standard- and reverse-phase encoded blipped non-diffusion-weighted images were also obtained to correct for the echo-planar imaging distortion correction (Andersson et al., 2016).

Acquisition parameters were as follows: repetition time, 9810 ms; echo time, 100 ms; voxel size, 2.0 × 2.0 × 2.0 mm^3^; matrix: 128 × 128; slices, 65; number of excitations, 1; and acquisition time, 13.07 min. T1-weighted images (T1WI) were also acquired for structural data by three-dimensional (3D) magnetization-prepared rapid gradient-echo sequence. The acquisition parameters were: repetition time, 15 ms; echo time, 3.54 ms; inversion time, 1100 ms; voxel size, 0.86 × 0.86 × 0.86 mm^3^; and acquisition time, 5.14 min.

### 2.3 Data preprocessing

**Fig. 1:**
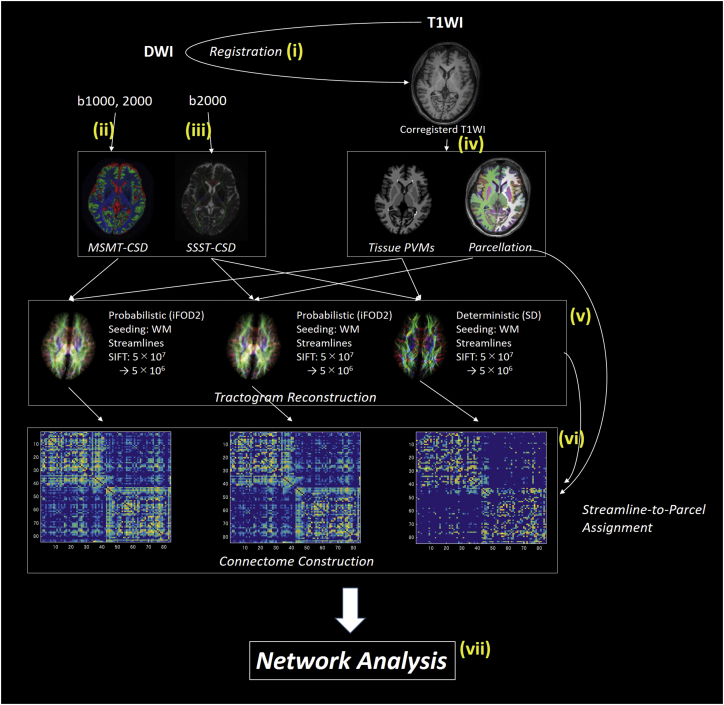
Schematic of structural network mapping. Structural brain networks were mapped according to the following sequence: (i) registration of T1WIs to the DW-MRIs; (ii) estimation of FODs using MSMT-CSD; (iii) estimation of FODs using SSST-CSD; (iv) estimation of tissue partial volume maps, parcellation of cortical and subcortical GM; (v) reconstruction of streamline tractogram using MSMT-CSD, CSD and deterministic tractography; (vi) construction of structural connectomes; (vii) structural brain network analysis. Abbreviations: CSD, constrained spherical deconvolution; GM, gray matter; iFOD2; a second-order integration over FOD algorithm; MSMT, multi-shell multi-tissue; SD, spherical deconvolution; SSST, single-shell single-tissue; WM, white matter.

Fig. 1 shows a schematic of the connectome mapping methodology. Preprocessing was performed using the Functional MRI of the Brain (FMRIB) Software Library, Version 5.0.9 (Greve and Fischl, 2009). For each subject, anatomical 3D–T1WIs were affine-aligned to the corresponding b0 maps using boundary-based registration. Partial volume fraction maps of WM, cortical GM, deep GM, and CSF, calculated from the T1WI data and then were processed for the multi-shell MSMT-CSD and anatomically-constrained tractography (ACT) framework. This involved the following steps.

First, the Brain Extraction Tool (Smith, 2002) was used to remove non-brain tissue from the 3D-T1WIs. Second, the FMRIB Automated Segmentation Tool (Zhang et al., 2001) estimated the partial volume fractions of WM, cortical GM, and CSF. Third, the FMRIB Integrated Registration and Segmentation Tool (Patenaude et al., 2011) were used to estimate partial volume fractions of the deep GM for all voxels within the brain. Fourth, the GM–WM interface mask was obtained using the “5tt2gmwmi” command, which was implemented in the MRtrix (http://www.mrtrix.org/). Although WM masks are commonly used for seeding, over-reconstruction of streamline density for longer fiber pathways can be induced by homogeneously seeding streamlines throughout the WM (Yeh et al., 2016). In this study, we used the GM–WM interface as a seeding point for tractogram generation, as it is reported that this method can avoid the over-reconstruction problem (Girard et al., 2014); however, this approach can result in an underestimation of the prevalence of long-distance fibers (Zalesky and Fornito, 2009).

DW-MRI data were checked visually in all three orthogonal views, which did not reveal severe artifacts related to gross geometric distortion, signal dropout, or bulk motion. The data were corrected for susceptibility-induced geometric distortions, eddy currents, and inter-volume motion using the TOPUP and EDDY toolboxes (Andersson and Sotiropoulos, 2016).

### 2.4 Node definition

A default reconstruction pipeline was performed in FreeSurfer (Dale et al., 1999) to obtain 84 brain nodes according to Desikan–Killiany cortical atlas segmentation (Desikan et al., 2006). Due to high variability in the spatial location and extent of subcortical GM segmentations produced by FreeSurfer (Dale et al., 1999), the subcortical structures were obtained from subcortical GM partial volume fraction maps using the FMRIB Integrated Registration and Segmentation Tool in the FMRIB Software Library (see the Data preprocessing section).

### 2.5 Edge definition

Whole-brain tractograms were generated using 1) deterministic SSST-CSD tracking (b-value of 2000 s/mm^2^), (2) probabilistic SSST-CSD tracking (b-value of 2000 s/mm^2^), and (3) probabilistic MSMT-CSD probabilistic tracking from multi-shell DW-MRI data (b-values of 0, 1000, and 2000 s/mm^2^). For all three methods (deterministic SSST-CSD tracking, probabilistic SSST-CSD tracking and probabilistic MSMT-CSD probabilistic tracking), spherical deconvolution informed filtering of tractograms (Smith et al., 2013) and ACT (Smith et al., 2012) were applied to reduce bias in streamline density and to prevent biologically unrealistic connection terminations (Smith et al., 2012). Tractography for all three methods was performed with the MRtrix software package (Brain Research Institute, Melbourne, Australia, http://www.brain.org.au/software/).

#### 2.5.1 Response function and fODF estimation for deterministic and probabilistic SSST-CSD tracking

For conventional CSD and deterministic tractography, voxels were assigned to WM if the tissue segment WM volume fraction was > 0.95 and the fractional anisotropy (FA) was > 0.7. Subsequently, the DW-MRI signal was reoriented to ensure that the principal axis of diffusion was aligned. Finally, the anisotropic response functions for WM were estimated from single shell data (b = 2000) by averaging the DW-MRI profiles over these voxels. The WM fODF was obtained using the dwi2fod command with the msmt-csd option, using WM response function. The maximal SH order lmax = 6 for WM was used. We generated probabilistic tractography (CSD) and deterministic tractography using these WM fODFs.

#### 2.5.2 Response function and fODF estimation for probabilistic MSMT-CSD tracking

For MSMT-CSD (Jeurissen et al., 2014), multiple response functions were estimated as a function of b-value and tissue type. Specifically, voxels were assigned to WM if the tissue segment WM volume fraction based on the structural image was > 0.95 and the fractional anisotropy (FA) was > 0.7. Subsequently, the DW-MRI signal was reoriented to ensure alignment of the principal axis of diffusion. Finally, the anisotropic response functions for WM were estimated per shell by averaging the DW-MRI profiles over these voxels. If a tissue segmentation had a volume fraction > 95% and an FA < 0.2, voxels were assigned to GM and CSF. The response functions for GM and CSF were obtained by averaging DW-MRI profiles per shell. Finally, the WM fODF, GM fODF, and CSF fODF were obtained using the dwi2fod command with the msmt-csd option. A maximal spherical harmonic (SH) order of lmax = 6 for WM, lmax = 0 for GM and CSF were used. The WM-fODFs obtained here were used for probabilistic MSMT-CSD tracking.

#### 2.5.3 Fiber tracking

Data with a b-value of 2000 s/mm^2^ were used for deterministic and probabilistic SSST-CSD tracking, which is the basic requirement for ODF reconstruction of high angular resolution diffusion imaging (Pichon et al., 2005). In addition, data with b-values of 1000 and 2000 s/mm^2^ were used for probabilistic MCMT-CSD tracking.

For deterministic SSST-CSD tracking, we employed a deterministic algorithm based on spherical deconvolution (SD) (Tournier et al., 2012), referred to as SD_STREAM in MRtrix software, with the following parameters: step size = 0.2 mm, maximum curvature = 45° per step, length = 4–200 mm, and fiber orientation distributions threshold = 0.1.

For probabilistic SSST-CSD and MSMT-CSD tracking, second-order integration was employed over the FOD (iFOD2) algorithm (Tournier et al., 2010), using the following parameters: step size, 1.0 mm; maximum curvature, 45° per step; length, 4–200 mm; and fiber orientation distribution threshold, 0.06 for probabilistic MSMT-CSD tracking, 0.1 for deterministic and probabilistic SSST-CSD tracking.

For all tracking methods, in total, 5 × 10^7^ streamlines were seeded from WM fODFs. Seed points were determined dynamically using the Spherical deconvolution informed filtering of tractograms (SIFT) model (Smith et al., 2013). Furthermore, “back-tracking” was used within the ACT framework (Smith et al., 2012). SIFT was also applied to filter the reconstruction from 5 × 10^7^ to 5 × 10^6^ streamlines.

### 2.6 Connectome construction

Connectomes were constructed for each subject based on connectivity derived from the three tractography methods (i.e., deterministic SSST-CSD tracking, probabilistic SSST-CSD tracking and probabilistic MSMT-CSD probabilistic tracking). WM connectivity was modeled as a weighted, undirected network (connectome). Nodes were defined as the 84 brain regions of the Desikan–Killiany GM parcellation, and the number of streamlines interconnecting each pair of nodes was enumerated. Specifically, streamlines were assigned to the closest node within a 2 mm radius of each streamline endpoint in ACT (Smith et al., 2015). This resulted in an 84 × 84 interregional connectivity matrix, with each element populated by the number of streamlines that served as a measure of connectivity strength. The diagonal elements represented self-connections and were excluded from analysis. A connection density threshold (T) was applied to discard spurious links (Rubinov and Sporns, 2010). Pairs of regions with the lowest streamline counts were set to a value of zero, and the top T% of regions according to streamline count were left unaltered. To avoid bias from using a single threshold, global network metrics were examined across a range of thresholds (10% < T < 30% in 5% increments) (Zhang et al., 2011).

### 2.7 Graph theory analyses

Topological measures were analyzed for the three sets of connectivity matrices, as derived from deterministic SSST-CSD tracking, probabilistic SSST-CSD tracking and probabilistic MSMT-CSD probabilistic tracking, using the Brain Connectivity Toolbox (http://www.brain-connectivity-toolbox.net/). The following five global network metrics were computed: global path length, clustering coefficient, global efficiency, mean strength, and the small-worldness ratio. In addition, four local network metrics were computed: nodal strength, betweenness centrality, local clustering, and local efficiency. Detailed descriptions of the global and local network metrics are summarized in Supporting information Table S1. To estimate effect sizes, Cohen's *d* was computed for each global network metric, across the range of thresholds to discard spurious links (10% < T < 30% in 5% increments) for comparison between PD and control groups. Cohen's *d* was typically largest for a threshold of 30% (Supporting information Table S2), so this was applied as the threshold for both global and local network metrics.

### 2.8 Identification of disrupted WM connections

The network-based statistic (NBS) was used to identify subnetworks (clusters of nodes and edges) comprising connections with a reduced streamline count in patients with PD. The NBS was separately applied to the connectivity matrices derived from the three methods being compared (i.e., deterministic SSST-CSD tracking, probabilistic SSST-CSD tracking and probabilistic MSMT-CSD probabilistic tracking). A detailed description of NBS can be found elsewhere (Zalesky et al., 2010). In brief, a two-sample *t*-test was independently performed at each edge to test the null hypothesis of equality in mean streamline count between patients and controls. A set of suprathreshold edges was defined by applying a primary component-forming threshold (e.g., *P* = 0.005, *t* = 2.97, two-tailed *t*-test) to the test statistic computed for each edge (results across different thresholds are reported in Supporting information Table S3). The statistical significance of each connected component was obtained with respect to an empirical estimate of the null distribution of maximal component sizes (5000 permutations), with the component size measured as the number of edges it comprised. We reported any components that were significant at a *P*-value of 0.05 after family-wise error correction.

**Fig. S1:**
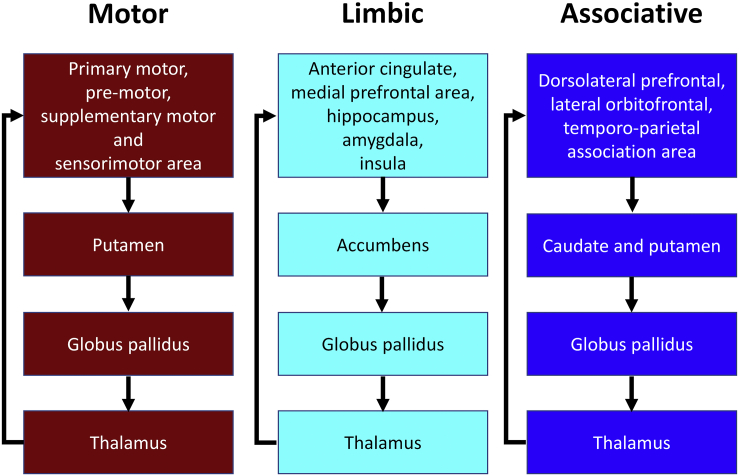

Connections comprising significant components (subnetworks) were assigned to motor, associative, or limbic circuits that constitute the cortico–basal ganglia–thalamocortical network (Galvan et al., 2015, Ikemoto et al., 2015). Further details are provided in Supporting information Fig. S1.

### 2.9 Classification of diagnostic status

A support vector machine (SVM) classifier was trained to predict an individual's diagnostic status (case or control), based on topological measures derived from the three tractography methods (i.e., deterministic SSST-CSD tracking, probabilistic SSST-CSD tracking and probabilistic MSMT-CSD probabilistic tracking). A linear kernel was used to train the SVM. The feature space comprised five global measures and two local measures (local clustering and local efficiency) from eight regions of interest. The selected regions of interest were subcortical structures spanning the cortico–basal ganglia–thalamocortical circuit (Galvan et al., 2015), including the putamen, globus pallidus, caudate, and thalamus. Local efficiency and clustering were used to measure the efficiency with which these regions, and by extension, the circuit of interest, could exchange neural information.

To train the SVM and to evaluate classification accuracy, we used stratified ten-fold cross-validation. The patient and control groups were partitioned into validation and training subgroups. The validation subgroup comprised 10% of all individuals in either the patient or control group (i.e., two controls and two patients), and all other individuals were randomly assigned to the training subgroup (i.e., 19 controls and 19 patients). The SVM was trained to classify patients and controls in the training subgroup based on latent patterns among the topological features described above. The validation subgroup was then used to evaluate the classification accuracy of the SVM. This process was repeated for ten unique validation subgroups (i.e., ten folds), with each individual only ever assigned to one validation subgroup. Classification performance was then averaged over these ten folds.

### 2.10 Statistical analysis

All statistical analyses were performed using IBM SPSS for Windows, Version 22.0 (IBM Corp., Armonk, NY, USA). Demographic and clinical variables were normally distributed, as confirmed with the Kolmogorov–Smirnov test. Between-group differences were analyzed by Student's *t*-tests for continuous variables (age, global, and local topological metrics) and by chi-squared tests for gender. Pearson's correlation coefficient was used to test for relationships between brain measures (e.g., connectivity strength and topological metrics) exhibiting significant between-group differences and clinical measures (e.g., disease duration and UPDRS-III score). The false discovery rate (FDR) was used to correct for multiple comparisons, using a significance threshold of *P* < 0.05.

## 3 Results

### 3.1 Disrupted WM connections

NBS did not identify any between-group differences when applied to connectivity matrices from deterministic SSST-CSD tracking. For both probabilistic SSST-CSD and MSMT-CSD tracking, the null hypothesis of equality in mean streamline count between patients with PD and controls was rejected (*P* < 0.05) for networks involving the basal ganglia, thalamic, limbic, frontal, temporal, and parietal areas (Fig. 4, Supporting information Table S4). The streamline count was reduced in the PD group relative to the control group. Specifically, probabilistic SSST-CSD tracking identified a comparable subnetwork of reduced connectivity comprising 44 edges connecting 43 regions (*P* = 0.007). These included 15 associative edges, 1 limbic edge, and 5 motor edges. By contrast, probabilistic MSMT-CSD tracking identified a significant subnetwork comprising 58 edges that connected 45 regions (*P* = 0.006) with a reduced streamline count in patients with PD. These included 17 edges classified as associative, 3 as limbic, and 7 as motor circuits. In addition, significant correlations were not detected between disease severity and the streamline count averaged across the subnetworks associated with significant between-group differences, although a trend involving the UPDRS-III score was evident for probabilistic MSMT-CSD tracking (Supporting information Table S5).

### 3.2 Global metrics

No between-group differences were found in any global metrics derived with deterministic SSST-CSD tracking. Probabilistic SSST-CSD tracking only detected significant differences in global connectivity strength and small-worldness, which were significantly decreased in patients with PD compared with healthy controls (Table 2). There were significant between-group differences across the five global measures with probabilistic MSMT-CSD tracking. Specifically, patients with PD displayed significantly decreased global connectivity strength, efficiency, and clustering, as well as increased global path length (Table 2). In addition, although both patients and controls demonstrated small-world organization (σ > 1), the small-worldness ratio was significantly decreased in the PD group. No correlations were found between global network metrics and clinical measures (e.g., the UPDRS-III score and disease duration) in the PD group.

**Table 2:** Between-group comparison of global network measures.

		Controls	Patients with PD	*t*	*P*⁎⁎	*d*⁎⁎⁎
Global clustering	Deterministic SSST-CSD (SD)	0.024 (0.006)	0.021 (0.004)	1.78	0.21	0.56
	Probabilistic SSST-CSD (SD)	0.013 (0.003)	0.011 (0.003)	2.23	0.053	0.70
	Probabilistic MSMT-CSD (SD)	0.018 (0.004)	0.015 (0.003)	2.33	0.036⁎	0.74
Global efficiency	Deterministic SSST-CSD (SD)	0.039 (0.006)	0.038 (0.009)	0.45	0.655	0.14
	Probabilistic SSST-CSD (SD)	0.036 (0.008)	0.031 (0.010)	1.73	0.094	0.55
	Probabilistic MSMT-CSD (SD)	0.044 (0.010)	0.038 (0.008)	2.15	0.037⁎	0.68
Global strength	Deterministic SSST-CSD (SD)	2738.62 (291.67)	2328.66 (831.24)	2.08	0.21	0.66
	Probabilistic SSST-CSD (SD)	3899.52 (232.29)	3501.76 (629.64)	2.65	0.025⁎	0.84
	Probabilistic MSMT-CSD (SD)	6442.84 (643.74)	5821.95 (808.86)	2.69	0.025⁎	0.85
Characteristic path length	Deterministic SSST-CSD (SD)	4.47 (0.31)	4.55 (0.56)	− 0.54	0.655	− 0.17
	Probabilistic SSST-CSD (SD)	4.69 (0.22)	4.84 (0.33)	− 1.72	0.094	− 0.54
	Probabilistic MSMT-CSD (SD)	4.55 (0.19)	4.70 (0.24)	− 2.27	0.036⁎	− 0.72
Small-worldness ratio	Deterministic SSST-CSD (SD)	3.05 (0.54)	2.64 (1.21)	1.39	0.286	0.44
	Probabilistic SSST-CSD (SD)	2.18 (0.49)	1.69 (0.65)	2.70	0.024⁎	0.85
	Probabilistic MSMT-CSD (SD)	4.44 (1.38)	3.01 (1.24)	3.45	0.005⁎	1.09

Notes: Data are expressed as mean (SD). Abbreviations: CSD, constrained spherical deconvolution; MSMT-CSD; multi-shell multi-tissue CSD; SD, standard deviation, SSST-CSD; single-shell single-tissue CSD.

⁎ Denotes statistical significance.

⁎⁎ *P*-values are corrected for the false discovery rate.

⁎⁎⁎ Cohen's *d*.

### 3.3 Local metrics

**Fig. 2:**
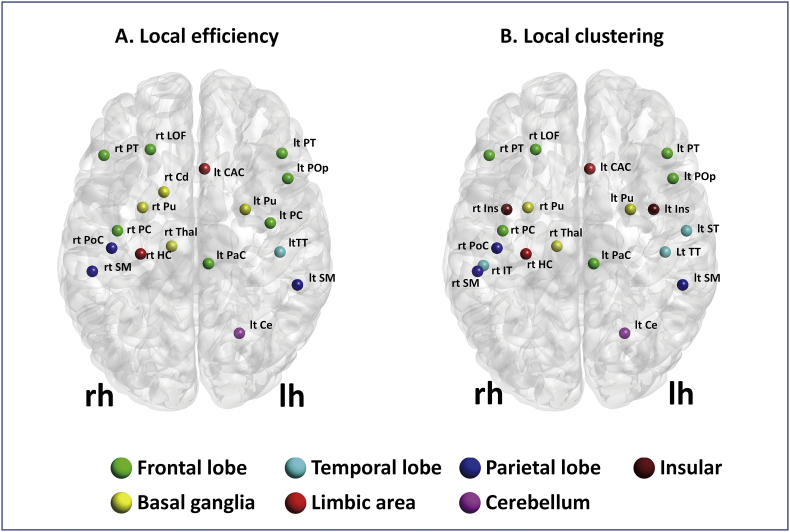
Local metrics results. (A) Regions with decreased local efficiency in patients with PD, as compared to healthy controls (*P* < 0.025, FDR corrected). (B) Regions with decreased local clustering in patients with PD as compared to healthy controls (*P* < 0.025, FDR corrected). Probabilistic MSMT-CSD was the only algorithm that detected significant between-group differences in local topological measures. Local efficiency and clustering were reduced in patients with PD in several regions, including the motor area, frontal temporoparietal associative area, limbic area, basal ganglia, and thalamus. Abbreviations: lh, left hemisphere; rh, right hemisphere; CAC, caudal anterior cingulate gyrus; Cd, caudate; Ce, cerebellum; FDR, false discovery rate; HC, hippocampus; LOF, lateral orbitofrontal; MSMT-CSD, multi-tissue constrained spherical deconvolution; PaC, paracentral gyrus; PC, precentral gyrus; PD, Parkinson's disease; PoC, postcentral gyrus; PoP, pars opercularis; PT, pars triangularis; Pu, putamen; SM, supramarginal gyrus; Thal, thalamus; TT, transverse temporal gyrus.

**Fig. 3:**
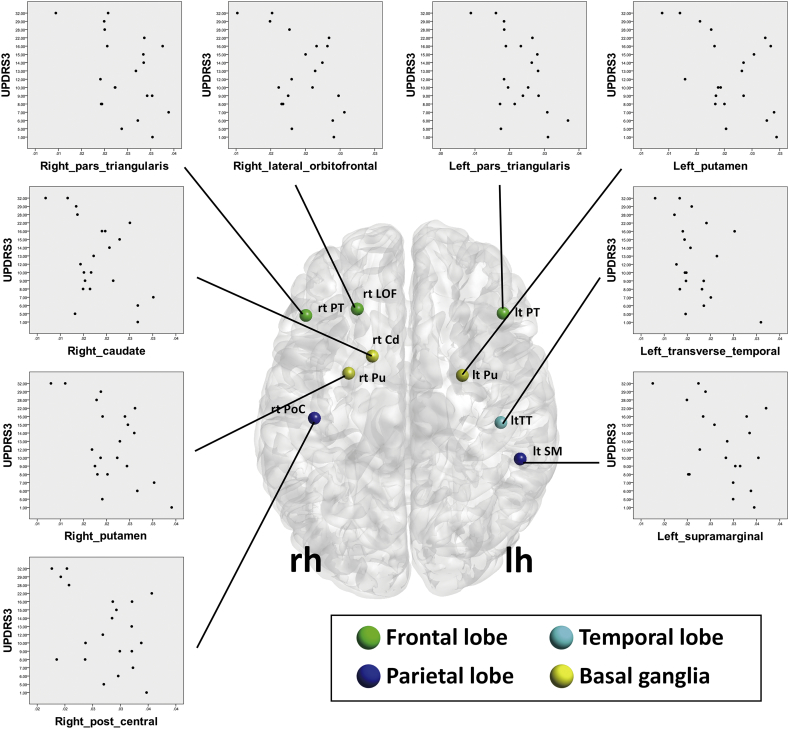
Regions where local efficiency were significantly correlated with the unified Parkinson's Disease Rating Scale (UPDRS)-III-motor subscale scores in the PD group. Abbreviations: lh, left hemisphere; rh, right hemisphere; Cd, caudate; LOF, lateral orbitofrontal; PoC, postcentral gyrus; PT, pars triangularis; Pu, putamen; SM, supramarginal gyrus; TT, transverse temporal gyrus.

Only probabilistic MSMT-CSD tracking detected significantly decreased local efficiency, predominantly localized to motor, frontal temporoparietal associative, limbic, basal ganglia, and thalamic areas (Fig. 2A, Table 3). The PD group also displayed reduced local efficiency, most evidently in the motor, frontal temporoparietal associative, limbic, basal ganglia, and thalamic areas (Fig. 2B, Table 4). Significant negative correlations were observed between UPDRS-III scores and local efficiency in the bilateral putamen, bilateral pars triangularis, right caudate nucleus, right lateral orbitofrontal gyrus, right post central gyrus, left supramarginal gyrus, left transverse temporal gyrus (Fig. 3, Table 5). This correlation was mainly seen in the motor area, frontal temporoparietal associative area, and basal ganglia, and no significant correlations were detected between local metrics and disease duration.

**Fig. 4:**
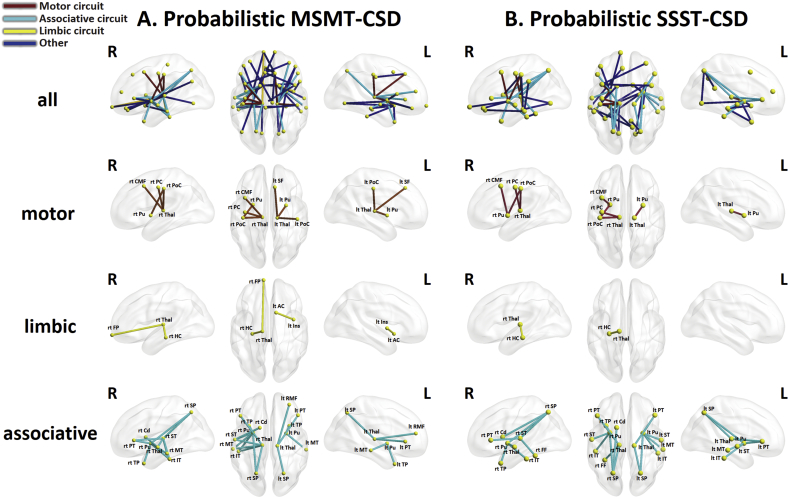
Subnetworks for which the streamline count was significantly reduced in patients with PD relative to healthy controls. (A) Result of probabilistic MSMT-CSD. (B) Result of probabilistic SSST-CSD. The first row shows all connections comprising subnetworks associated with significant between-group differences. The second, third, and fourth rows show these connections stratified according to motor, limbic, and associative circuits, respectively. Abbreviations: L, left hemisphere; R, right hemisphere; AC, accumbens; Cd, caudate; CMF, caudal middle frontal gyrus; CSD, constrained spherical deconvolution; FP, frontal pole; FF, fusiform gyrus; HC, hippocampus; Ins, insular; IT, inferior temporal gyrus; MSMT, multi-shell multi-tissue; MT, middle temporal gyrus; PC, precentral gyrus; PoC, postcentral gyrus; PT, pars triangularis; Pu, putamen; RMF, rostral middle frontal gyrus; SF, superior frontal gyrus; SP, superior parietal gyrus; SSST, single-shell single-tissue; ST, superior temporal gyrus; Thal, thalamus; TP, temporal pole.

**Table 3:** Regions with a significant between-group difference in local efficiency.

Regions	Controls	Patients with PD	FDR corrected	FDR-corrected *P* value	Cohen's *d*
Right putamen	0.028 (0.007)	0.021 (0.006)	3.49	0.041⁎	1.10
Right thalamus	0.031 (0.006)	0.024 (0.006)	3.46	0.041⁎	1.09
Right postcentral gyrus	0.034 (0.008)	0.027 (0.005)	3.26	0.041⁎	1.03
Left putamen	0.027 (0.007)	0.021 (0.006)	3.20	0.041⁎	1.01
Left paracentral	0.034 (0.009)	0.026 (0.007)	3.01	0.041⁎	0.95
Left cerebellum	0.033 (0.007)	0.026 (0.008)	2.95	0.041⁎	0.93
Right caudate	0.024 (0.006)	0.018 (0.006)	2.94	0.041⁎	0.93
Left pars triangularis	0.030 (0.009)	0.023 (0.006)	2.92	0.041⁎	0.92
Left transverse temporal gyrus	0.021 (0.004)	0.017 (0.005)	2.91	0.041⁎	0.92
Right supramarginal gyrus	0.033 (0.008)	0.027 (0.005)	2.90	0.041⁎	0.92
Right pars triangularis	0.030 (0.007)	0.024 (0.006)	2.83	0.041⁎	0.89
Left caudal anterior cingulate gyrus	0.027 (0.009)	0.021 (0.005)	2.83	0.041⁎	0.89
Right lateral orbitofrontal gyrus	0.024 (0.006)	0.020 (0.004)	2.82	0.041⁎	0.89
Right precentral gyrus	0.044 (0.009)	0.036 (0.009)	2.81	0.041⁎	0.89
Left supramarginal gyrus	0.033 (0.007)	0.027 (0.006)	2.81	0.041⁎	0.89
Left precentral gyrus	0.042 (0.009)	0.034 (0.008)	2.80	0.041⁎	0.89
Right hippocampus	0.025 (0.006)	0.021 (0.004)	2.74	0.043⁎	0.87
Left pars opercularis	0.029 (0.007)	0.023 (0.006)	2.74	0.043⁎	0.87

Notes: Data are expressed as mean (SD). Abbreviations: CSD, constrained spherical deconvolution; FDR, false discovery rate; MSMT-CSD, multi-shell multi-tissue CSD; SD, standard deviation.

⁎ Statistical significance.

**Table 4:** Regions with a significant between-group difference in local clustering.

Regions	Controls	Patients with PD	*t*	*P* value⁎⁎	Cohen's *d*
Right supramarginal gyrus	0.020 (0.005)	0.015 (0.003)	3.39	0.032⁎	1.07
Right insula	0.012 (0.003)	0.009 (0.002)	3.25	0.032⁎	1.03
Right postcentral gyrus	0.022 (0.005)	0.018 (0.004)	3.24	0.032⁎	1.02
Right thalamus	0.014 (0.003)	0.011 (0.003)	3.23	0.032⁎	1.02
Left pars triangularis	0.020 (0.003)	0.014 (0.005)	3.21	0.032⁎	1.02
Left paracentral gyrus	0.023 (0.006)	0.017 (0.005)	3.15	0.032⁎	0.99
Right putamen	0.012 (0.003)	0.009 (0.002)	3.14	0.032⁎	0.99
Left supramarginal gyrus	0.021 (0.005)	0.016 (0.004)	3.08	0.032⁎	0.97
Left caudal anterior cingulate gyrus	0.017 (0.005)	0.013 (0.003)	3.04	0.032⁎	0.96
Right lateral orbitofrontal gyrus	0.014 (0.004)	0.011 (0.003)	3.03	0.032⁎	0.96
Right pars triangularis	0.020 (0.005)	0.016 (0.004)	3.01	0.032⁎	0.95
Left pars opercularis	0.017 (0.004)	0.013 (0.004)	3.00	0.032⁎	0.95
Right precentral gyrus	0.024 (0.005)	0.020 (0.005)	2.97	0.033⁎	0.94
Left transverse temporal gyrus	0.018 (0.003)	0.014 (0.004)	2.94	0.033⁎	0.93
Left superior temporal gyrus	0.012 (0.003)	0.010 (0.002)	2.88	0.034⁎	0.91
Right inferior temporal gyrus	0.018 (0.003)	0.014 (0.004)	2.85	0.034⁎	0.90
Left insula	0.011 (0.003)	0.009 (0.002)	2.84	0.034⁎	0.90
Left putamen	0.011 (0.003)	0.009 (0.002)	2.83	0.034⁎	0.90
Left cerebellum	0.023 (0.005)	0.018 (0.006)	2.73	0.041⁎	0.86
Right hippocampus	0.012 (0.003)	0.010 (0.002)	2.67	0.046⁎	0.84

Notes: Data are expressed as mean (SD). Abbreviations: CSD, constrained spherical deconvolution; FDR, false discovery rate; MSMT-CSD, multi-shell multi-tissue CSD; SD, standard deviation.

⁎ Statistical significance.

⁎⁎ *P*-values are corrected for the false discovery rate.

**Table 5:** Regions where local efficiency was significantly correlated with UPDRS-III.

Regions	FDR-corrected *P* value	*R*
Right putamen	0.033⁎	− 0.621
Left pars triangularis	0.033⁎	− 0.583
Left putamen	0.033⁎	− 0.572
Right lateral orbitofrontal gyrus	0.033⁎	− 0.545
Right post central gyrus	0.033⁎	− 0.543
Right caudate	0.036⁎	− 0.528
Left supramarginal gyrus	0.038⁎	− 0.516
Right pars triangularis	0.038⁎	− 0.508
Left transverse temporal gyrus	0.049⁎	− 0.483

Abbreviations: FDR, false discovery rate; UPDRS, Unified Idiopathic Parkinson's Disease Rating Scale.

⁎ Statistical significance.

### 3.4 Prediction of diagnosis

SVM based on probabilistic MSMT-CSD tracking yielded improved classification accuracies compared to deterministic and probabilistic SSST-CSD tracking (see Table 6). A feature space comprising all five global measures yielded the highest classification accuracy (78.33%), precision (85.00%), recall (81.67%), and area under the curve (85.28%). However, a feature space comprising only two local measures (i.e., clustering and efficiency) across eight regions (i.e., bilateral putamen, globus pallidus, caudate, and thalamus) yielded lower, but reasonable accuracy (61.67%), precision (46.67%), recall (51.67%), and area under the curve (68.06%). SVM based on probabilistic MSMT-CSD tracking with the five global and two local measures combined yielded modest accuracy (76.67%), precision (81.67%), recall (76.67%), and area under the curve (81.39%).

**Table 6:** Classifier performance for distinguishing between patients with PD and healthy controls.

Features set	Accuracy	SD of accuracy	Precision	Recall	AUC
All global metrics (deterministic SSST-CSD)	60.00%	25.50%	45.00%	35.00%	66.94%
All global metrics (probabilistic SSST-CSD)	56.67%	21.98%	50.00%	51.67%	41.94%
All global metrics (probabilistic MSMT-CSD)	78.33%	13.54%	85.00%	81.67%	85.28%
Local clustering and local efficiency (deterministic SSST-CSD)	50.00%	27.39%	45.00%	46.67%	43.33%
Local clustering and local efficiency (probabilistic SSST-CSD)	45.83%	17.97%	41.67%	48.33%	53.33%
Local clustering and local efficiency (probabilistic MSMT-CSD)	61.67%	11.90%	46.67%	51.67%	68.06%
All global metrics, local clustering, and local efficiency (deterministic SSST-CSD)	41.67%	24.15%	36.67%	36.67%	41.67%
All global metrics, local clustering, and local efficiency (probabilistic SSST-CSD)	57.50%	25.12%	60.00%	48.33%	71.67%
All global metrics, local clustering, and local efficiency (probabilistic MSMT-CSD)	76.67%	13.84%	81.67%	76.67%	81.39%

Abbreviations: AUC, area under the curve; CSD, constrained spherical deconvolution; MSMT, multi-shell, multi-tissue; PD, Parkinson's disease; SSST, single-shell single-tissue.

## 4 Discussion

We compared DW-MRI-based connectomes derived from three different algorithms (i.e., deterministic SSST-CSD tracking, probabilistic SSST-CSD tracking, and probabilistic MSMT-CSD tracking) between patients with idiopathic PD and healthy controls. Across all analyses, probabilistic MSMT-CSD tracking outperformed the deterministic and probabilistic SSST-CSD tracking methods when detecting connectome abnormalities and accurately predicting PD diagnosis. At the global level, probabilistic MSMT-CSD tracking detected significant between-group differences across all five global measures, while probabilistic SSST-CSD tracking only detected lower global strength and small-worldness in the PD group. At the local level, probabilistic MSMT-CSD tracking detected significantly reduced local efficiency and clustering in patients, indicating greater functional segregation in the motor, frontal temporoparietal associative, limbic, basal ganglia, and thalamic areas. NBS identified subnetworks of reduced connectivity from the cortico–basal ganglia–thalamocortical network in patients with PD via both the probabilistic SSST-CSD and MSMT-CSD tracking methods.

Our data suggest that probabilistic MSMT-CSD tracking provides improved sensitivity to the WM connectivity disruptions typically associated with PD, though it should be noted that we did not evaluate the specificity of this approach. The improved sensitivity of probabilistic MSMT-CSD tracking may be due to the use of more precise fODF estimates at the GM–WM interface (Jeurissen et al., 2014), which is essential for accurately characterizing both nodes (GM regions) and the edges between nodes. Although probabilistic SSST-CSD tracking could partially detect connectivity disruptions, deterministic SSST-CSD tracking could not detect significant between-group differences in topological measures or connectivity strength. This may be due to differences in the algorithms used for global tracking (e.g., edge generation). In the present study, we adopted a fODF-based deterministic tracking algorithm (deterministic SSST-CSD tracking). This tracking method provides fiber distributions that are thought to correspond more closely with known brain anatomy, compared to conventional deterministic tensor tracking algorithms (Jeurissen et al., 2011). However, the probabilistic fODF-based algorithm has been shown to provide more accurate tractography compared to the deterministic fODF-based algorithm (Tournier et al., 2012). Although deterministic tracking provides a single best fit streamline, probabilistic tracking algorithms can explore other possible directions to account for the uncertainty of fiber orientation distributions. Therefore, in many cases, probabilistic methods can identify peripheral branches of tracts, where these branches might be difficult to detect with deterministic tractography. However, an important disadvantage of probabilistic tractography is that its high sensitivity typically comes at the cost of low specificity; namely, there is an increased likelihood of identifying spurious fibers (false positives) with probabilistic methods (Knosche et al., 2015, Thomas et al., 2014). The estimation of spurious fibers is particularly detrimental to the characterization of the connectome's topological properties (Zalesky et al., 2016).

For probabilistic MSMT-CSD tracking, using stratified ten-fold cross-validation, we could predict an individual's diagnostic status with modest accuracy (approximately 78%) based on graph theory. The most accurate prediction of diagnostic status was achieved with all global measures computed in the probabilistic MSMT-CSD tracking connectivity matrices (78.33%). The global measures for deterministic and probabilistic SSST-CSD tracking performed substantially less well than those in probabilistic MSMT-CSD tracking, with no parameter yielding an accuracy of > 70%. Although diagnostic accuracy was low when we used local graph metrics of the basal ganglia and thalamus, diagnostic accuracy (61.67%) was highest for local graph metrics derived from probabilistic MSMT-CSD tracking. This provides further evidence of the increased sensitivity of probabilistic MSMT-CSD tracking for detecting connectome pathology compared with deterministic and probabilistic SSST-CSD tracking.

Although our findings indicate that PD can be predicted with modest accuracy by probabilistic MSMT-CSD tracking, further work is needed to establish whether the classifier performance is robust to alternative image acquisition sequences and alternative probabilistic tractography algorithms. Deep learning methods, decision trees, and other supervised learning methods appropriate for neuroimaging data might improve the achieved classification accuracies (Vieira et al., 2017). Indeed, a criticism of SVM is the need to extract less redundant and more informative data from the raw data during feature selection (Plis et al., 2014). In this study, we selected features based on subcortical regions spanning a circuit known to be affected by PD pathology. Deep learning methods can automatically identify the optimal representation of features within a high-dimensional dataset, without requiring prior feature selection. Longitudinal studies are now required to determine the accuracy with which machine learning techniques trained on probabilistic MSMT-CSD tracking data can predict patient outcome and prognosis.

In the graph theory analysis using probabilistic MSMT-CSD tracking, we found decreased global clustering, efficiency, strength, and small-worldness, as well as increased global path length in PD. Reduced global clustering suggests poor network segregation (i.e., reduced specialized information processing), whereas decreased global efficiency and increased global path length indicate compromised network integration (i.e., reduced parallel information transfer). These global, local, and connectivity alterations are consistent with the results of previous studies (Li et al., 2016, Nigro et al., 2016) and probably reflect the presence of extensive pathological changes in the WM of PD brains. In particular, the α-synuclein inclusions that are deposited in the pre-synapse of patients with PD may cause synaptic collapse and impaired axonal transport, resulting in widespread axonal degeneration (Braak and Del Tredici, 2008). Structural WM disintegration (reduced connectivity) can impair efficient information exchange, resulting in network disorganization (poor segregation and integration).

Previous connectome studies report unimpaired small-world organization (Li et al., 2016, Nigro et al., 2016). While we also found that small-world organization was maintained in PD (σ > 1), the small-world ratio measured by probabilistic MSMT-CSD tracking was decreased relative to that in controls. Previous studies have evaluated small-worldness by deterministic tracking based on the “Fiber Assignment by Continuous Tracking” algorithm (Li et al., 2016, Nigro et al., 2016), but this cannot accurately estimate neural fiber connections in regions with crossing and kissing fibers at the voxel level (Mori et al., 2004). By contrast, we applied probabilistic tracking that can deal with the crossing/kissing problem, and therefore yielded more accurate estimates of the connectome. Small-world organization is considered to reflect the optimal balance of functional integration and segregation, with the reduced small-world property in this study suggesting that this balance may be disrupted in PD.

At the local level, decreased segregation was found among key components of the cortico–basal ganglia–thalamocortical network, as indicated by decreased local efficiency and clustering. These changes were correlated with the UPDRS-III motor score, thereby strongly supporting involvement of this network in PD pathophysiology. Nigro et al. reported reduced local efficiency and clustering in the globus pallidus and inferior occipital gyrus (Nigro et al., 2016), but we found similar reductions across a more extensive area, including the motor, frontal temporoparietal associative, and basal ganglia areas. This difference might be due to discrepant connectome generation methods (as described above); however, discrepancies in disease durations between studies may be important. The participants in the study by Nigro et al. (2016) had early PD (mean duration of 1 year 7 months) and had not received medications, whereas participants in our study had a mean illness duration of 5 years and all had already received levodopa treatment. Therefore, pathological changes may have progressed to a wider area among the patients in our study.

Both probabilistic SSST-CSD and MSMT-CSD connectomes revealed subnetworks of reduced connectivity between key components of the cortico–basal ganglia–thalamocortical network. The results of NBS analysis also provide evidence supporting involvement of this network in PD pathophysiology. Other studies using NBS have reported reduced connectivity strength between the basal ganglia, thalamic, limbic, frontal, temporal, and parietal areas in patients with PD (Aarabi et al., 2015, Kim and Park, 2016, Li et al., 2016, Nigro et al., 2016), which is also in agreement with our results.

This study has some limitations: First, the sample size was small and the study design was retrospective in nature. Larger, multicenter, prospective studies are therefore required. In addition, we only included patients with relatively long durations of PD, so a longitudinal study of prodromal or early PD is required to confirm whether connectome analyses based on probabilistic MSMT-CSD tracking is effective for monitoring and predicting disease progression. Second, we did not evaluate the non-motor symptoms of PD, such as cognitive and psychiatric symptoms. Because connectivity disruptions in the basal ganglia–thalamocortical circuits might contribute to non-motor symptoms in PD, future studies should investigate the relationship between non-motor symptoms and connectome dysfunction more closely. Finally, because the PD diagnoses were not confirmed histopathologically, misdiagnosis remains possible; however, the validity of each diagnosis was supported by continued response to therapy and continued freedom from atypical parkinsonism at 12 months after scanning.

## 5 Conclusion

We mapped the connectomes of patients with PD and healthy controls to determine which of deterministic SSST-CSD tracking, probabilistic SSST-CSD tracking, and probabilistic MSMT-CSD probabilistic tracking was most sensitive for detecting associated WM connectivity disruptions. Probabilistic MSMT-CSD tracking detected WM disruptions most extensively among the three methods, and these disruptions were characteristic of known PD pathophysiology, including connectivity loss in cortico–basal ganglia–thalamocortical networks. Moreover, when compared with deterministic and probabilistic SSST-CSD tracking connectome methods, probabilistic MSMT-CSD tracking more accurately classified patients with PD from healthy comparison subjects. We, therefore, conclude that connectome analysis based on probabilistic MSMT-CSD tracking offers greater sensitivity and accuracy when assessing aberrant WM connectivity in cases of suspected PD. However, further research is needed to confirm our results in larger studies, and specific research is needed to look at the specificity of probabilistic MSMT-CSD tracking in prodromal or early stages of PD when there is diagnostic uncertainty.

The following are the supplementary data related to this article.

Supportive information figure S1 Segregated basal ganglia—thalamocortical circuit. The motor circuit involves projections from the supplementary motor area, arcuate premotor area, motor cortex and somatosensory cortex into the putamen, which projects into the globus pallidus which projects into the cortex through the thalamus. Therefore, we classified connections linking these areas as motor circuit. The limbic circuit involving the projections from the anterior cingulate, hippocampus, entorhinal cortex, and insula into the ventral striatum, then into the globus pallidus, followed by a loop back into the cortex through the thalamus. Therefore, we classified connections linking these areas as limbic circuit. The associative circuit proposes a pathway from the dorsolateral prefrontal cortex, lateral orbitofrontal cortex, parietal and temporal association area into the caudate, putamen, followed by a projection into the globus pallidus, followed by a loop back into the cortex through the thalamus. Therefore, we classified connections linking these areas as cognitive/associative circuit. (based on Galvan et al., 2015). Image 1
