# Supplementary material for: Connectome analysis with diffusion MRI in idiopathic Parkinson's disease: Evaluation using multi-shell, multi-tissue, constrained spherical deconvolution
Source: Neuroimage Clin. 2017 Nov 10;17:518–29. doi: 10.1016/j.nicl.2017.11.007 (PMC5700829; doi:10.1016/j.nicl.2017.11.007)
Supplement: Supportive information figure S1 — Segregated basal ganglia—thalamocortical circuit. The motor circuit involves projections from the supplementary motor area, arcuate premotor area, motor cortex and somatosensory cortex into the putamen, which projects into the globus pallidus which projects into the cortex through the thalamus. Therefore, we classified connections linking these areas as motor circuit. The limbic circuit involving the projections from the anterior cingulate, hippocampus, entorhinal cortex, and insula into the ventral striatum, then into the globus pallidus, followed by a loop back into the cortex through the thalamus. Therefore, we classified connections linking these areas as limbic circuit. The associative circuit proposes a pathway from the dorsolateral prefrontal cortex, lateral orbitofrontal cortex, parietal and temporal association area into the caudate, putamen, followed by a projection into the globus pallidus, followed by a loop back into the cortex through the thalamus. Therefore, we classified connections linking these areas as cognitive/associative circuit. (based on Galvan et al., 2015). [file mmc1.docx]

**Table S1. Definitions and interpretations of graph metrics**

| **Metrics** | **Interpretation** |
| --- | --- |
| **Local measures** | |
| **Nodal strength** | The simplest measure of ***centrality*** (importance) of a given node. The nodal strength reflects a degree of interconnectivity with other regions (Watts and Strogatz, 1998). |
| **Betweenness centrality** | This also measures the ***centrality*** (importance) of a node, but reflects the importance of nodes in the information transfer within a network (Freeman, 1979). |
| **Local clustering** | This represents the ***functional segregation*** of a node, reflecting the degree of clustered connectivity around a given node (Rubinov and Sporns, 2010; Watts and Strogatz, 1998). |
| **Local efficiency** | This represents ***functional segregation*** of a node, which measures the capacity to transfer information among neighbors of that node (Rubinov and Sporns, 2010). |
| **Global measures** | |
| **Mean strength** | This is the average of the nodal strength of all nodes. |
| **Global clustering** | This is the average of the local clustering of all nodes and measures the ***functional segregation*** of a network. Global clustering reflects the prevalence of clustered connectivity around individual nodes (Rubinov and Sporns, 2010; Watts and Strogatz, 1998). |
| **Global efficiency** | This also measures the ***functional integration*** of a network, representing its capacity to exchange information efficiently (Rubinov and Sporns, 2010). |
| **Characteristic path length** | This is the most commonly used measure of ***functional integration,*** quantifying the ability to transfer information in parallel over the whole brain network (Rubinov and Sporns, 2010). |
| **Small-world property** | Small-world networks are defined as networks that are significantly more clustered than random networks, but where the characteristic path length between them is similar to that in random networks (Watts and Strogatz, 1998). A small-world property ($\sigma$) > 1 indicates that the network has small-world property (Humphries and Gurney, 2008). |

Freeman LC (1979): Centrality in social networks conceptual clarification. Soc Networks 1:215-239.

Humphries MD, Gurney K (2008): Network "small-world-ness": a quantitative method for determining canonical network equivalence. PloS one 3:e0002051.

Rubinov M, Sporns O (2010): Complex network measures of brain connectivity: uses and interpretations. NeuroImage 52:1059-1069.

Watts DJ, Strogatz SH (1998) Collective dynamics of "small-world" networks. Nature 393:440-442.

**Table S2. Cohen’s *d* of each global metric across the full range of sparsity thresholds for comparison between the PD and control groups**

| **Deterministic SSST-CSD tracking** | **Cohen’s *d*** | | | | |
| --- | --- | --- | --- | --- | --- |
| Metrics | 10% | 15% | 20% | 25% | 30% |
| Mean strength | 0.640 | 0.657 | 0.658 | 0.658 | 0.658 |
| Global clustering | 0.091 | 0.421 | 0.562 | 0.562 | 0.562 |
| Global efficiency | 0.140 | 0.142 | 0.142 | 0.142 | 0.142 |
| Characteristic path length | −0.104 | −0.172 | −0.172 | −0.172 | −0.172 |
| Small-world property | 0.317 | 0.441 | 0.421 | 0.437 | 0.440 |
| **Probabilistic SSST-CSD tracking** | **Cohen’s *d*** | | | | |
| Metrics | 10% | 15% | 20% | 25% | 30% |
| Mean strength | 0.718 | 0.765 | 0.778 | 0.785 | 0.838 |
| Global clustering | 0.593 | 0.675 | 0.643 | 0.702 | 0.704 |
| Global efficiency | 0.543 | 0.548 | 0.548 | 0.548 | 0.548 |
| Characteristic path length | −0.481 | −0.400 | −0.507 | −0.536 | −0.543 |
| Small-world property | 0.763 | 0.747 | 0.778 | 0.794 | 0.853 |
| **Probabilistic MSMT-CSD tracking** | **Cohen’s *d*** | | | | |
| Metrics | 10% | 15% | 20% | 25% | 30% |
| Mean strength | 0.782 | 0.822 | 0.837 | 0.846 | 0.849 |
| Global clustering | 0.631 | 0.725 | 0.746 | 0.730 | 0.740 |
| Global efficiency | 0.685 | 0.683 | 0.683 | 0.682 | 0.680 |
| Characteristic path length | −0.408 | −0.598 | −0.577 | −0.636 | −0.718 |
| Small-world property | 0.013 | 0.843 | 1.007 | 1.082 | 1.091 |

*Abbreviations:* CSD, constrained spherical deconvolution; MSMT, multi-shell, multi-tissue; PD, Parkinson’s disease; SSST, single-shell, single-tissue.

**Table S3: Networks identified as significantly different between patients with PD and Healthy Controls using Network-based Statistical Analysis**

|  | **Probabilistic MSMT-CSD tracking** | **Probabilistic SSST-CSD tracking** |
| --- | --- | --- |
| P-value = 0.05  T = 2.02 | **Network 1**  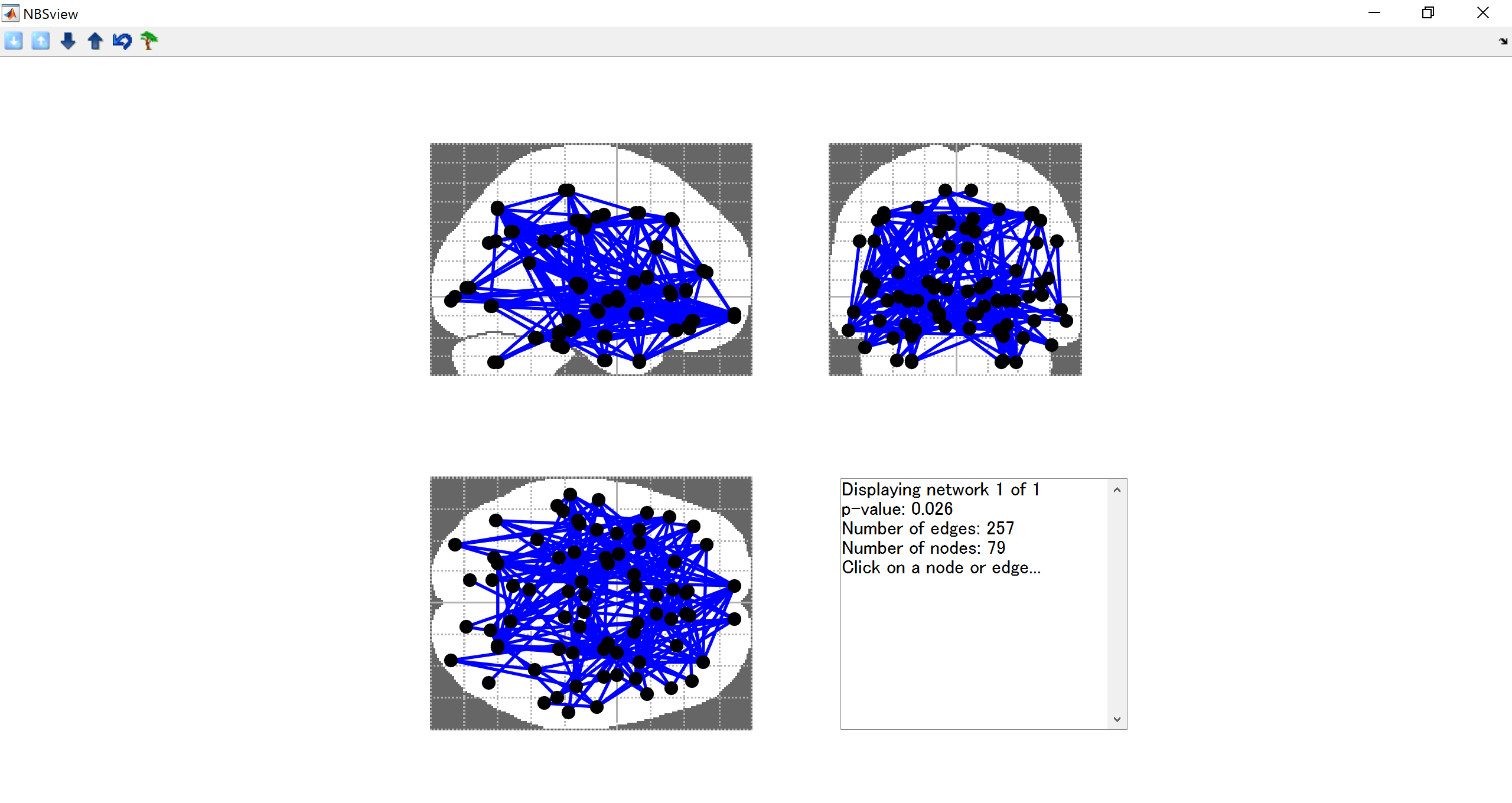 | **Network 1**  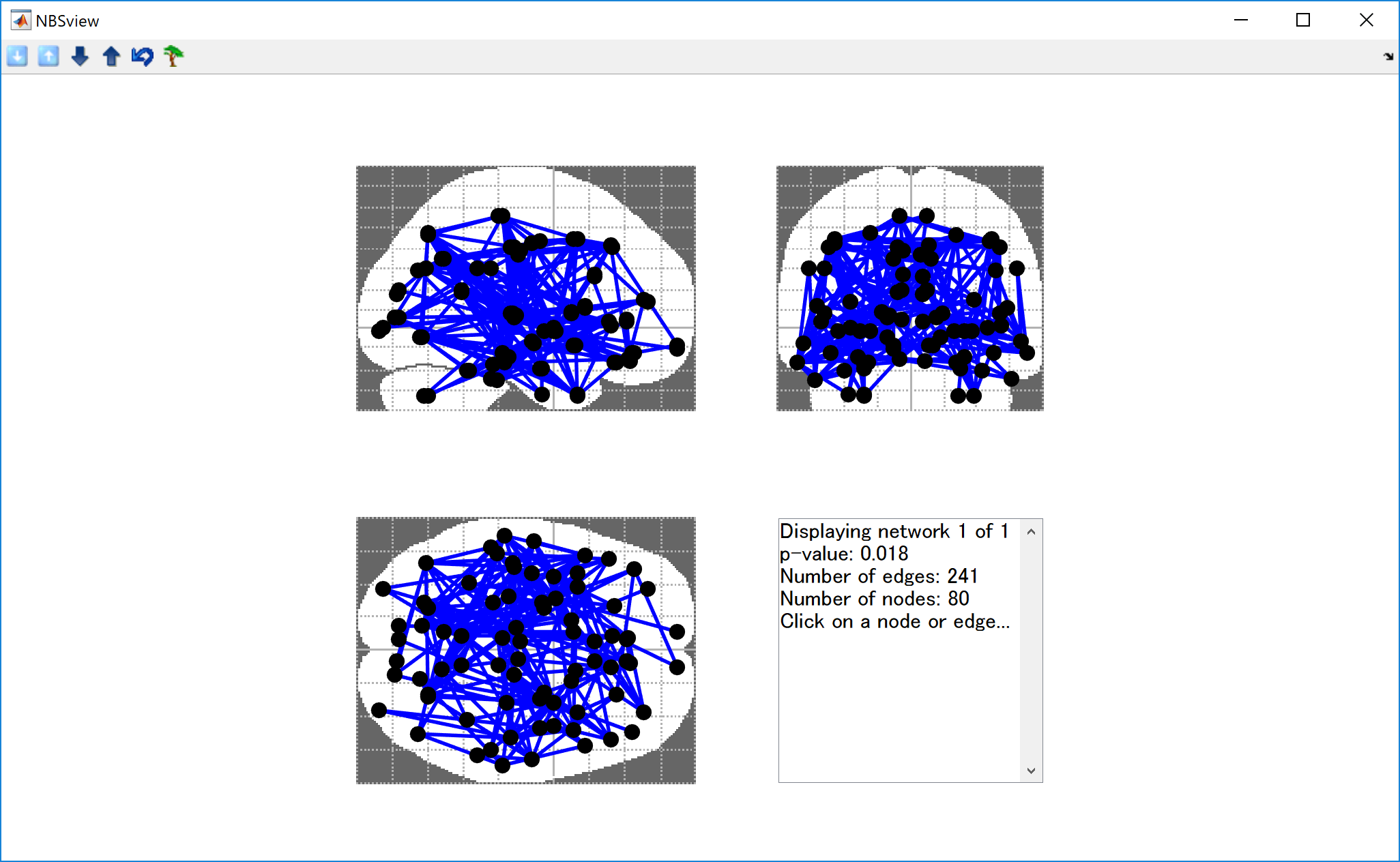 |
| P-value = 0.02  T = 2.42 | **Network 1**  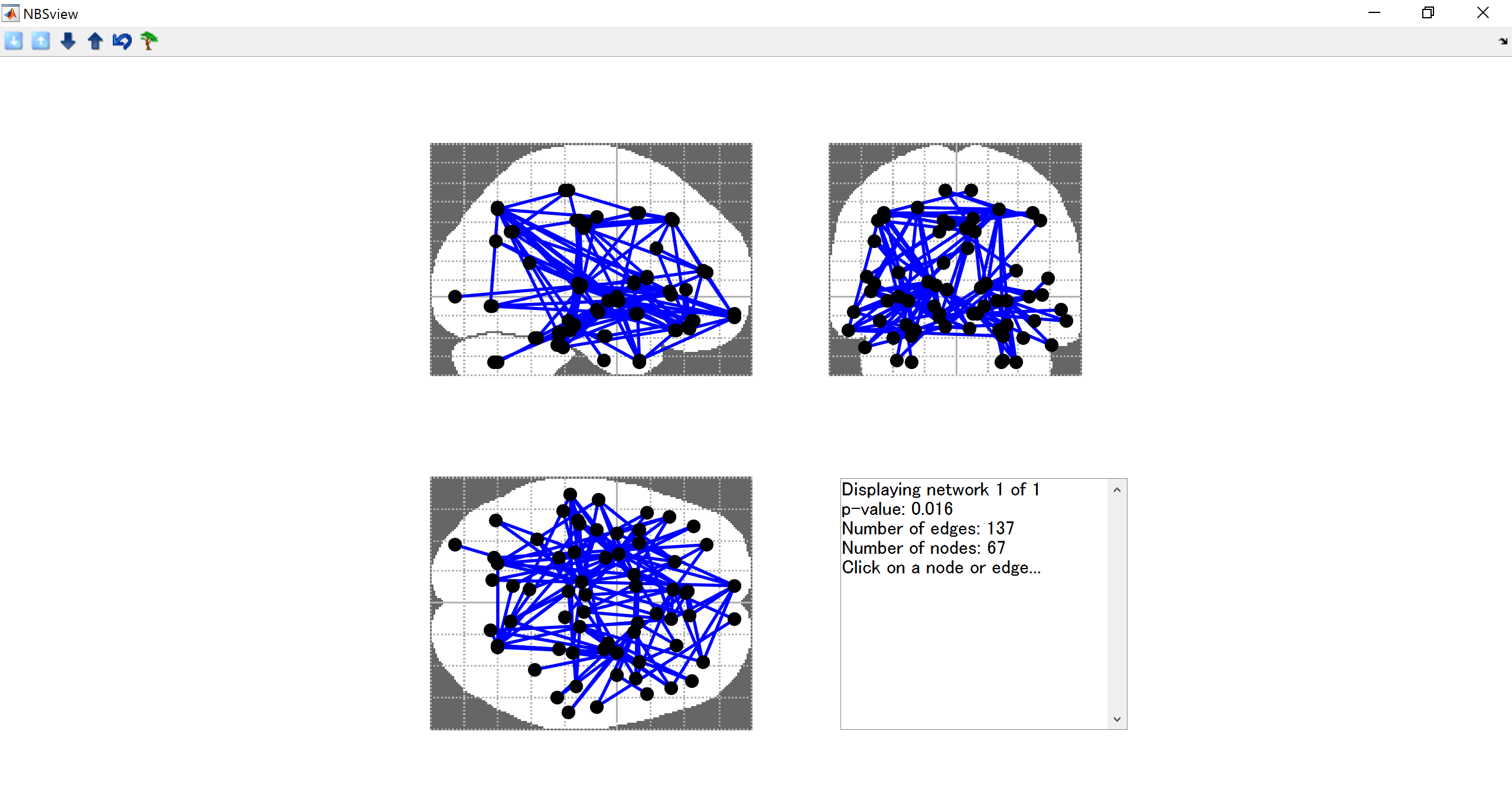 | **Network 1**  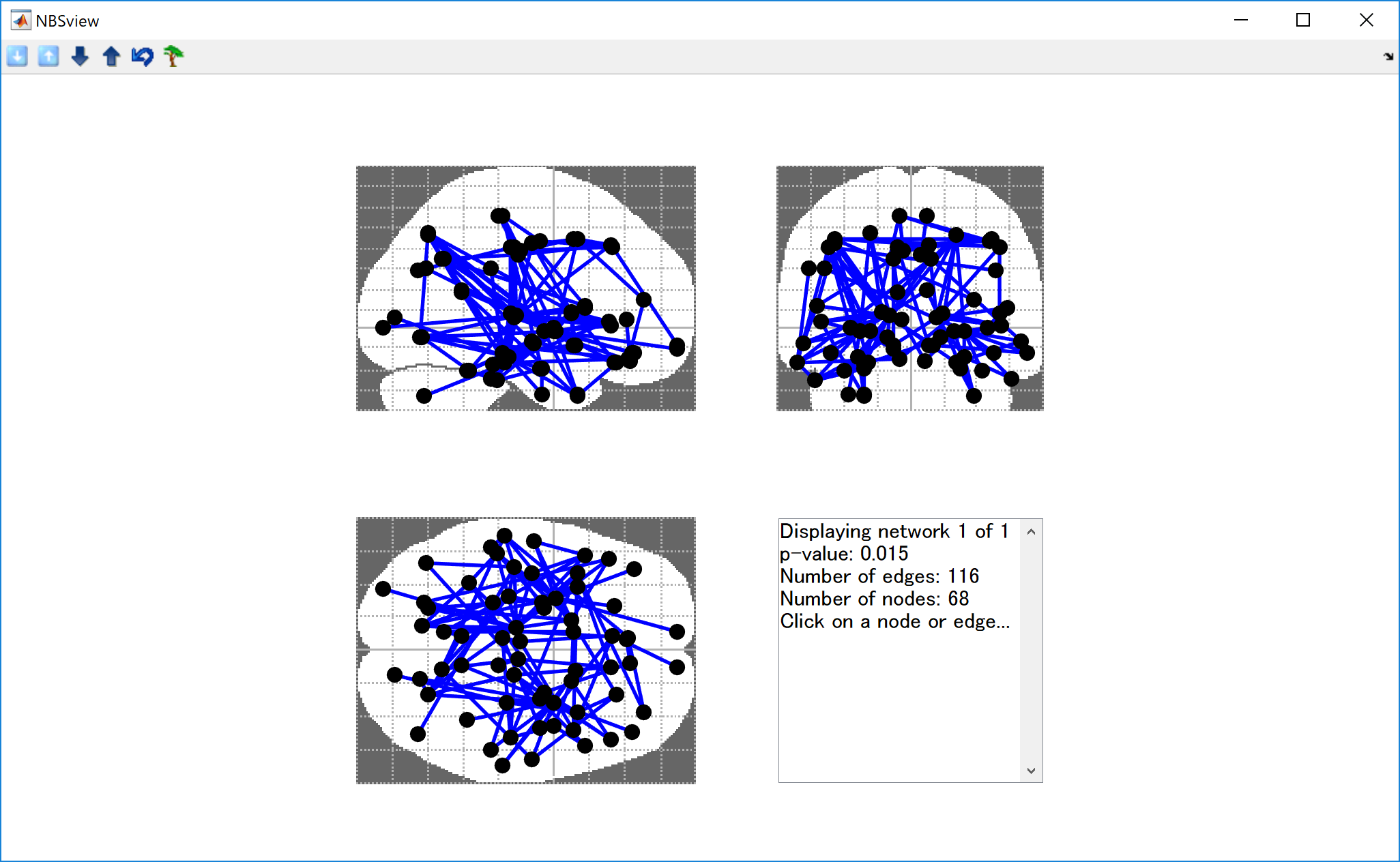 |
| P-value = 0.01  T = 2.70 | **Network 1**  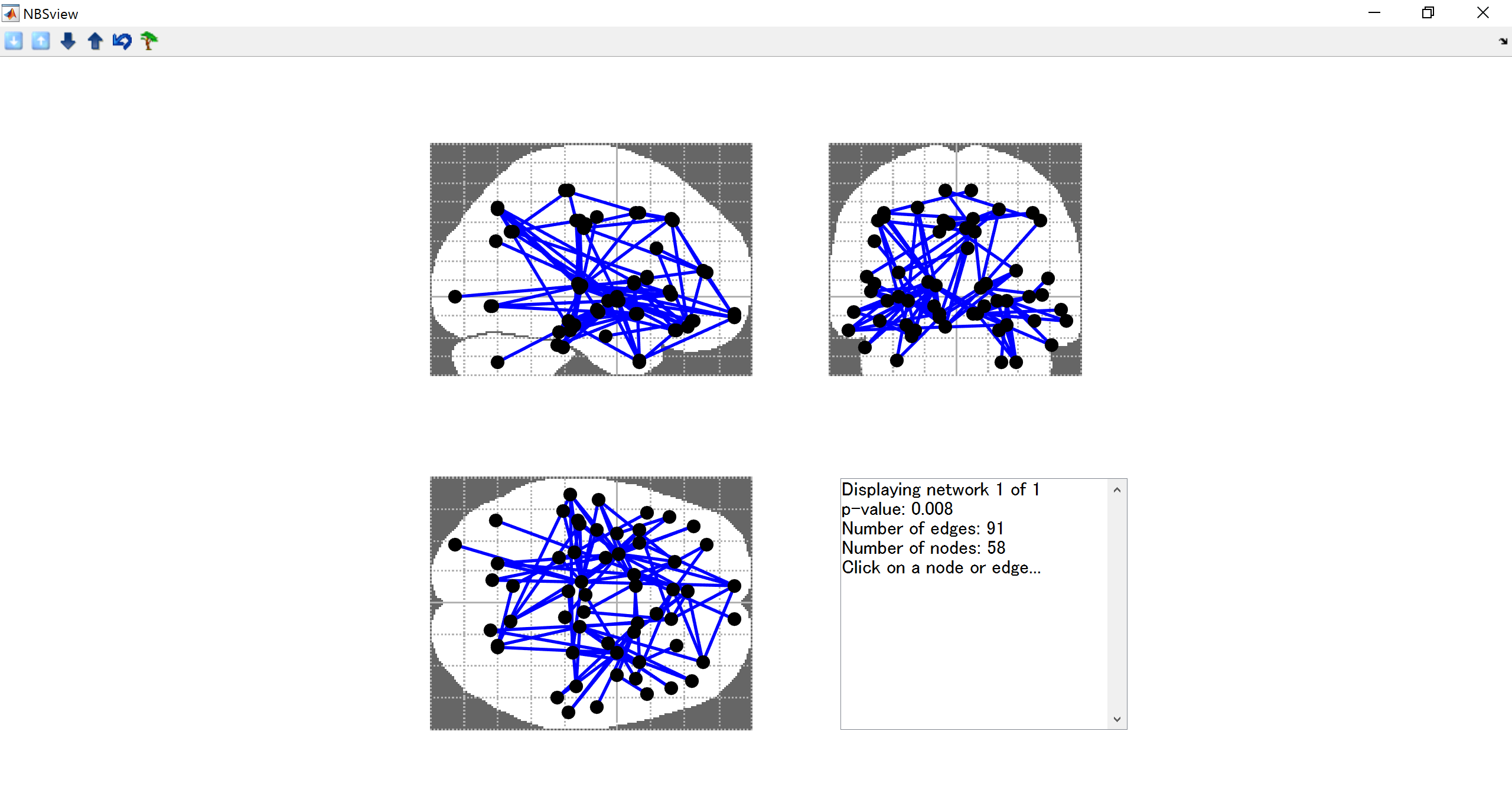 | **Network 1**  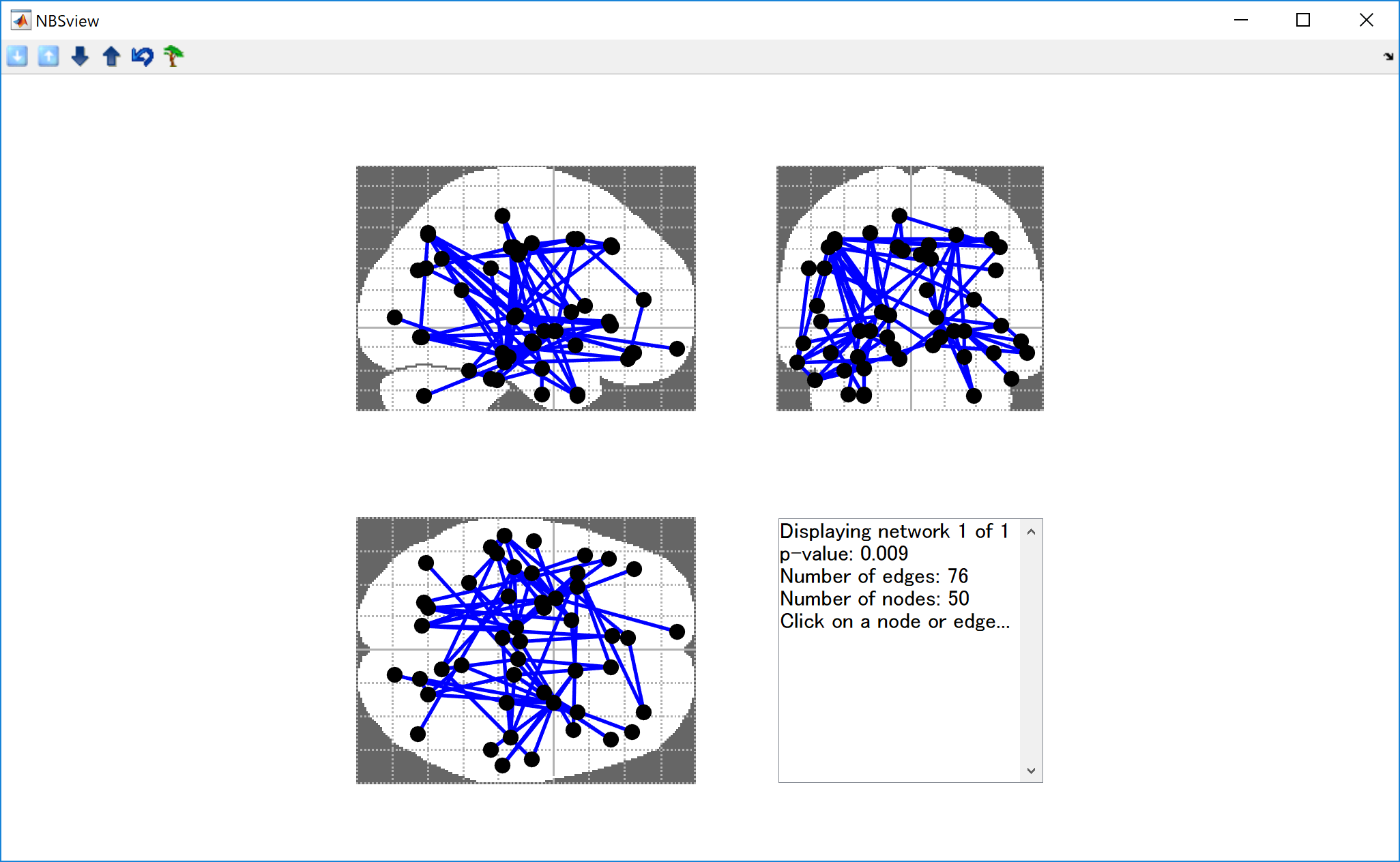 |
| P-value = 0.001  T = 3.54 | **Network 1**  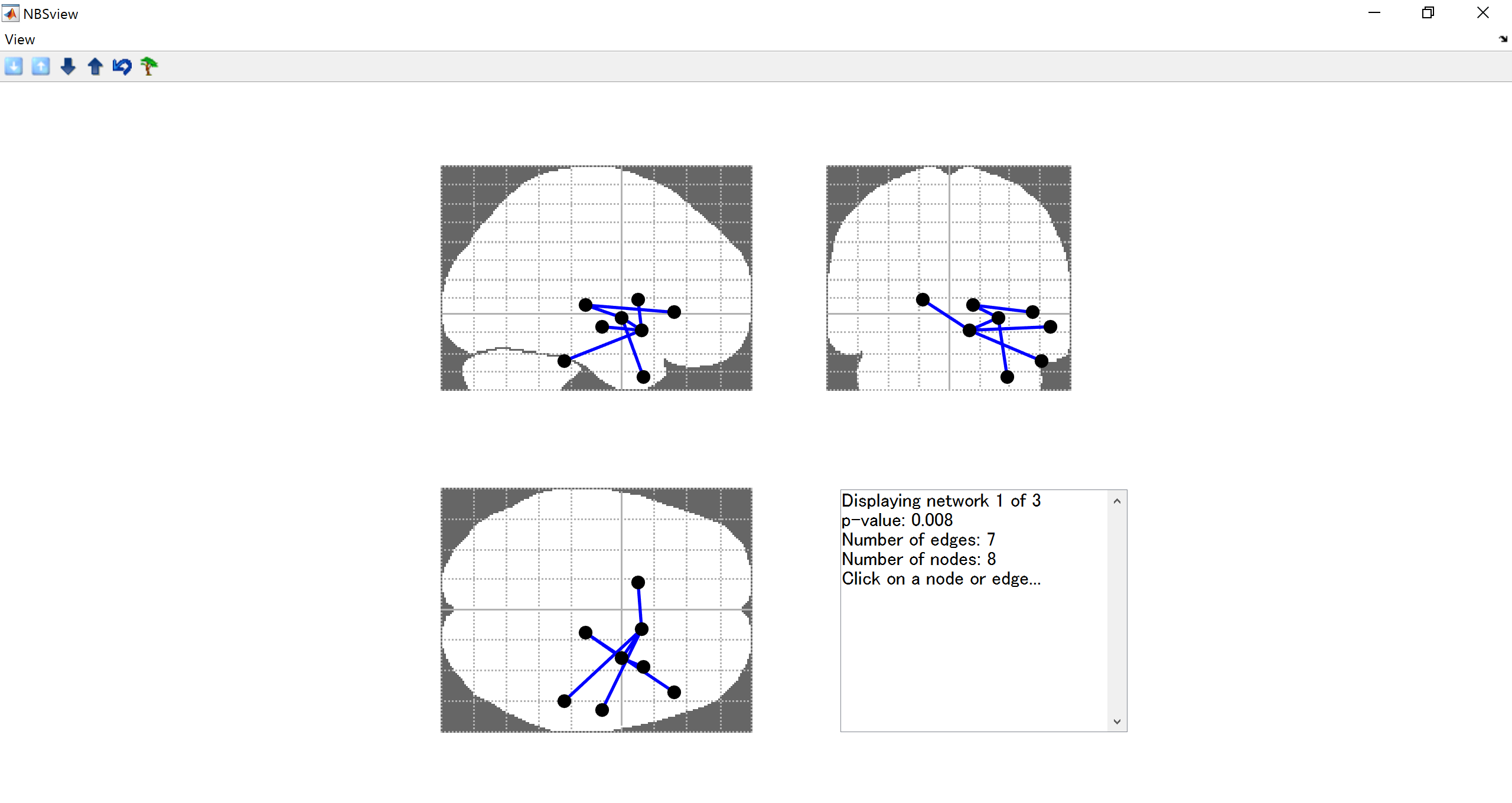  **Network 2**  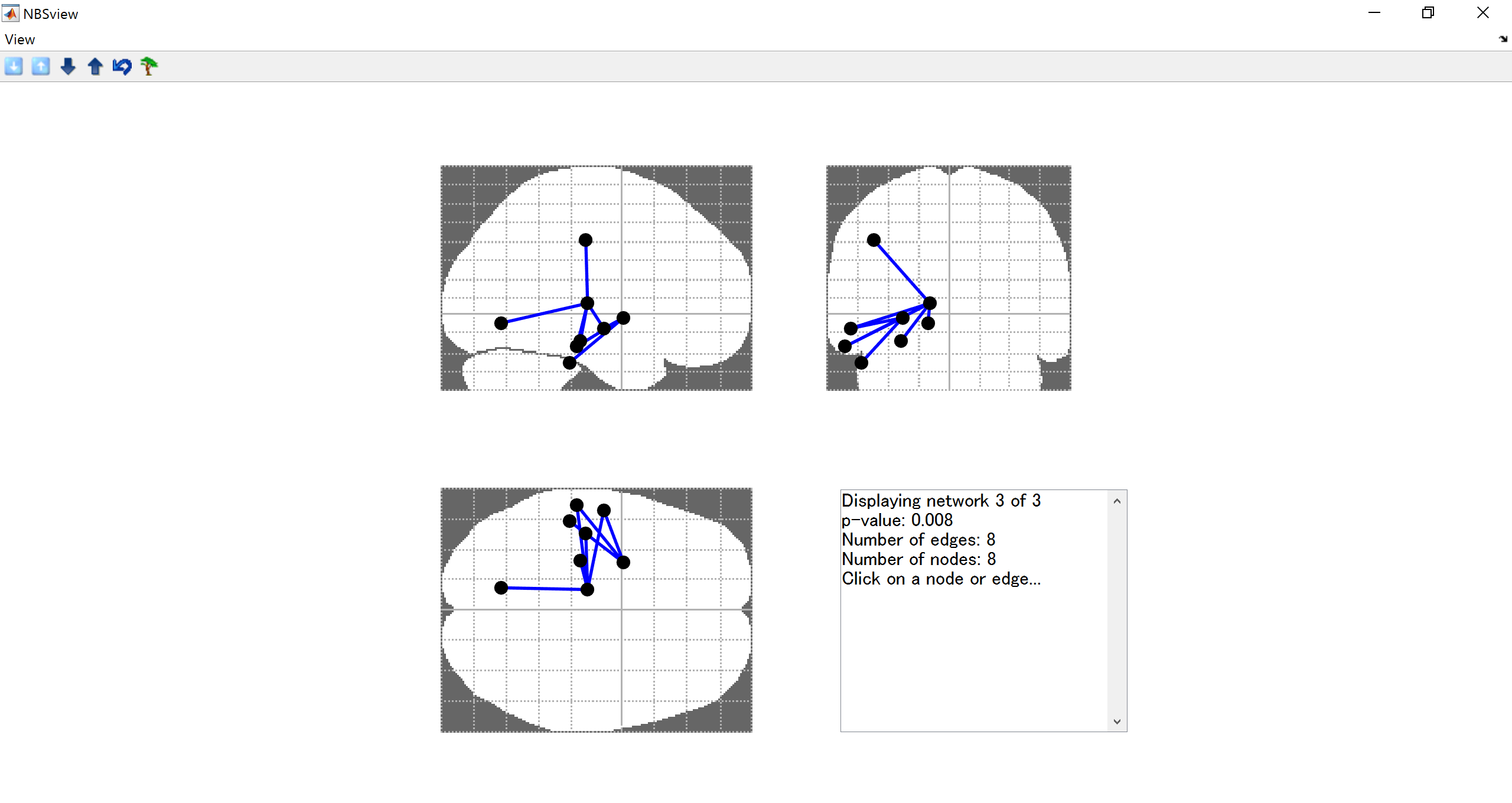  **Network 3**  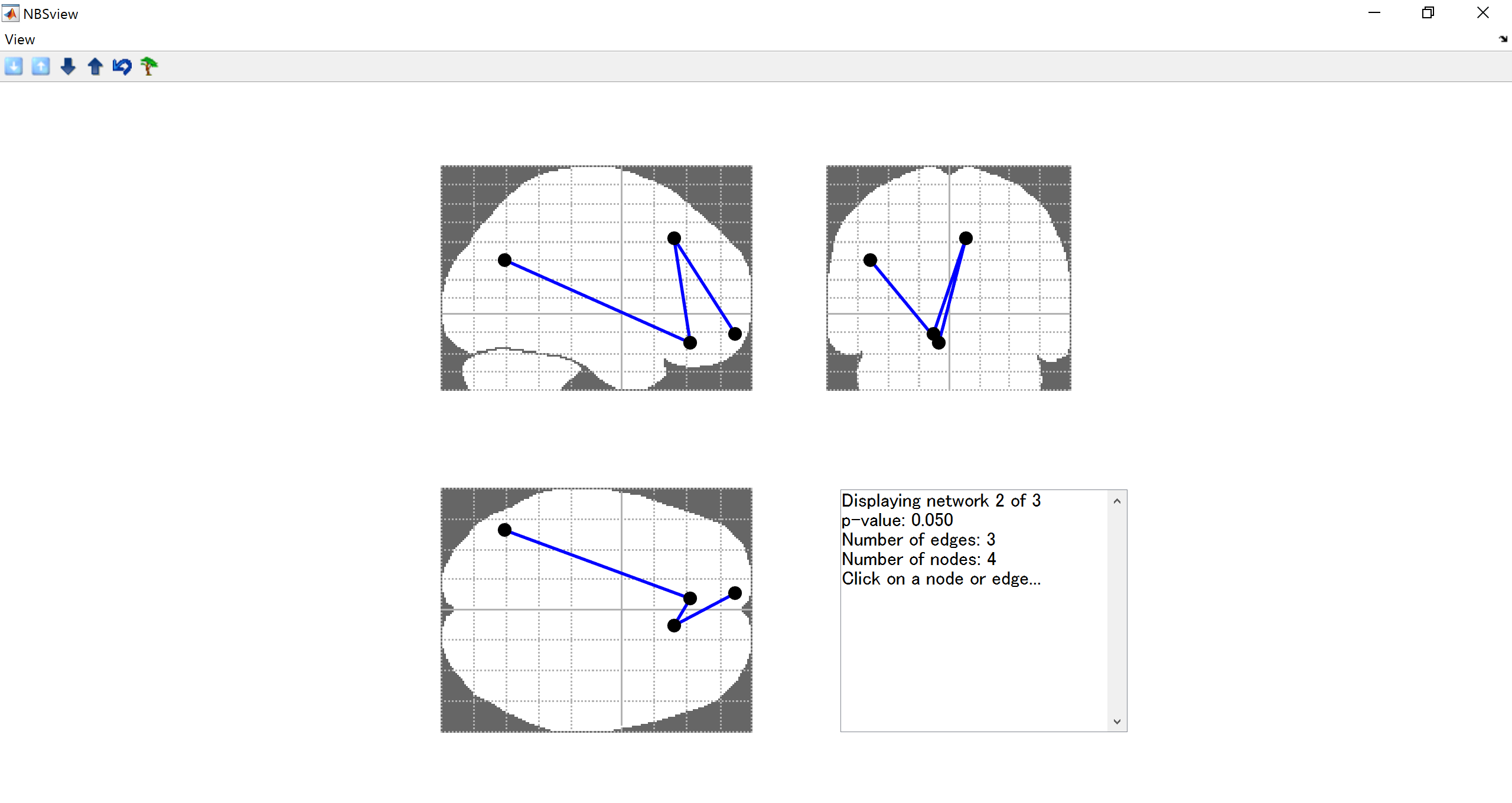 | **Network 1**  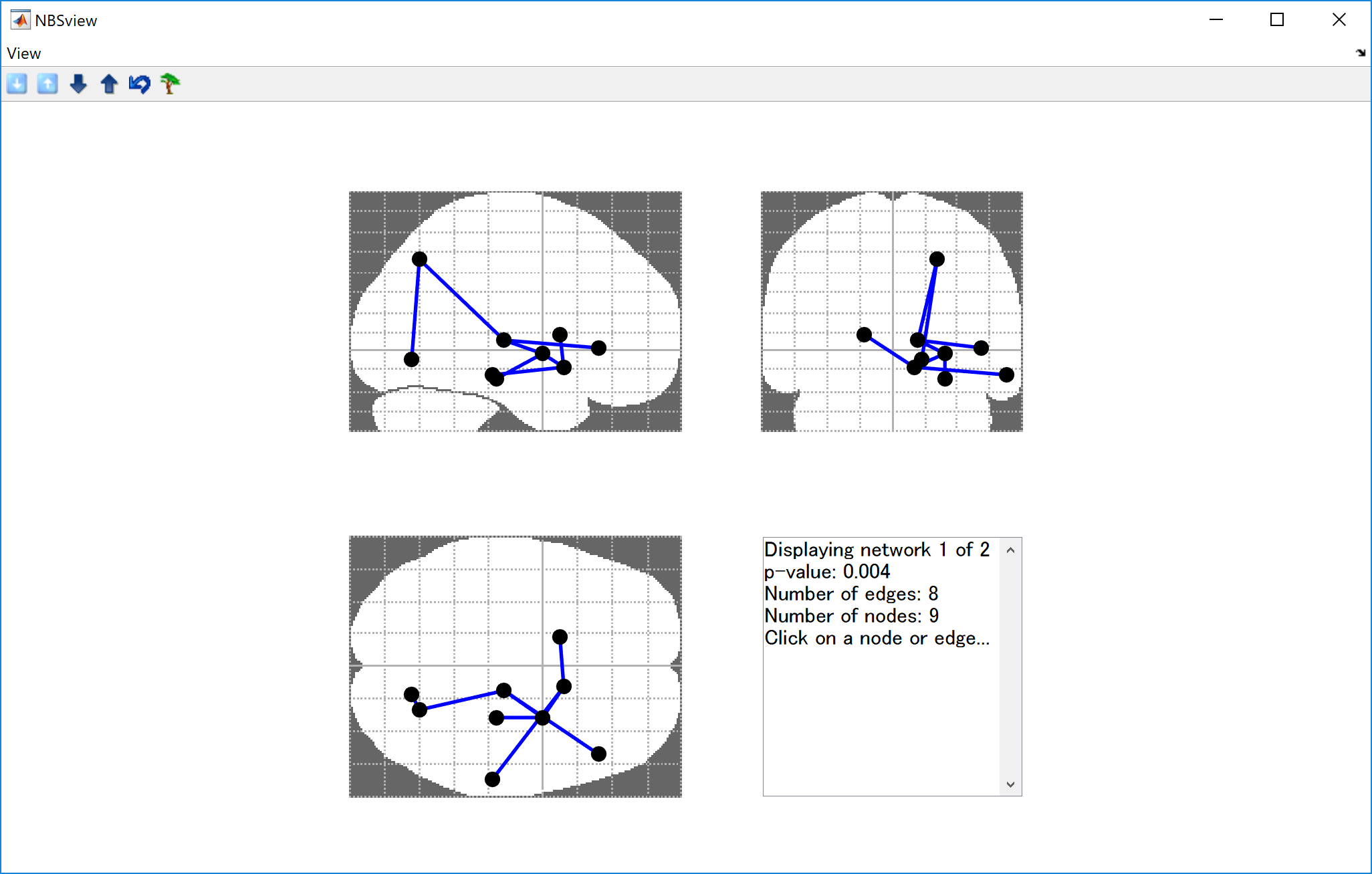  **Network 2**  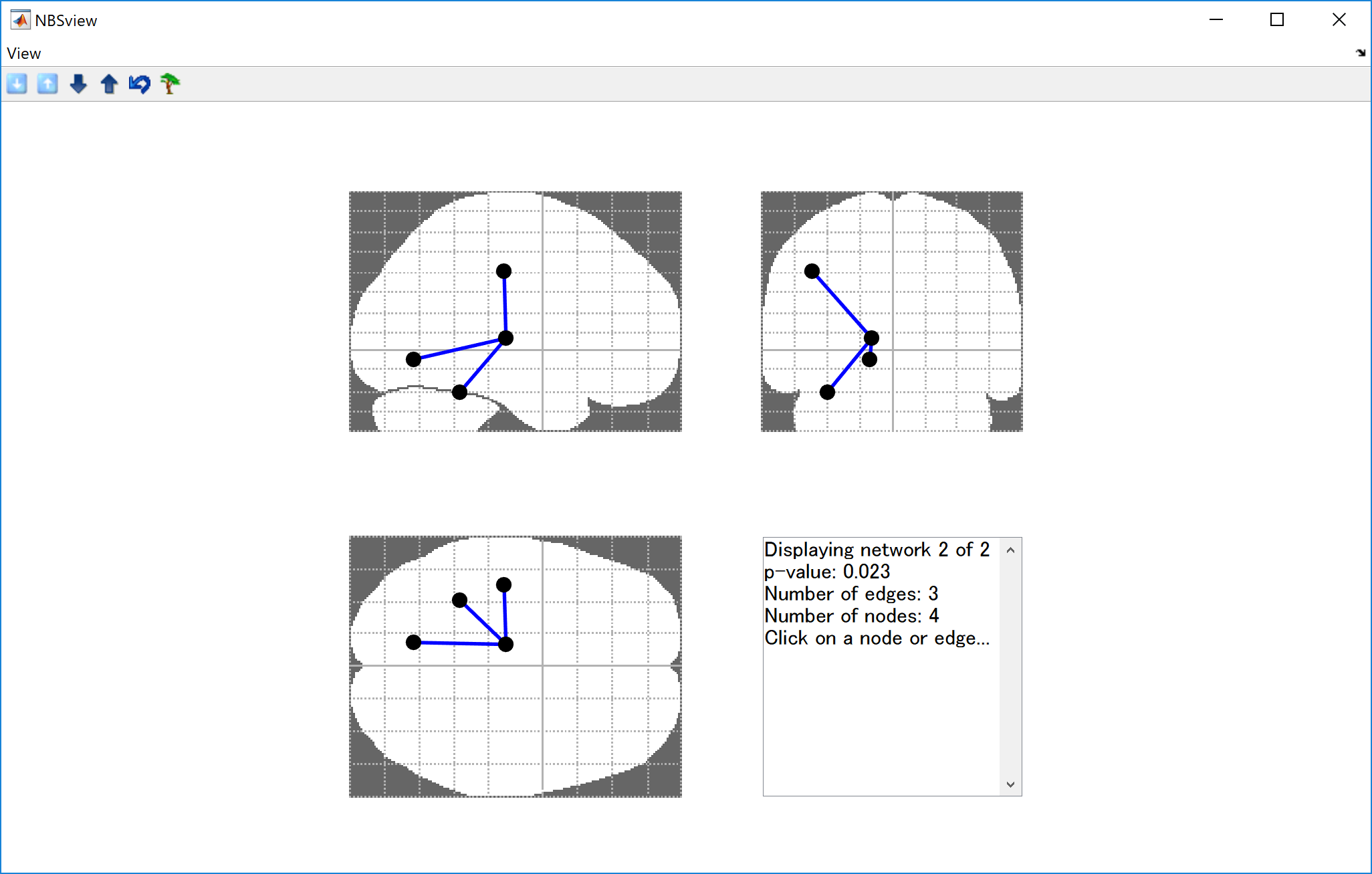 |

We explored the results of the network-based statistic, using different thresholds to individuate the suprathreshold edges (0.05 ≤ *P* ≤ 0.001). *Abbreviations:* CSD, constrained spherical deconvolution; MSMT, multi-shell, multi-tissue; PD, Parkinson’s disease; SSST, single-shell, single-tissue.

**Table S4: Networks identified as Significantly Different between Patients with PD and Healthy Controls using Network-Based Statistical Analysis**

| **Networks and Connections** | **t value** |
| --- | --- |
| **Network using probabilistic SSST-CSD (p = 0.007)** | |
| left-putamen to left-accumbens-area | 4.31 |
| left-accumbens-area to right-caudate | 4.07 |
| right-thalamus-proper to right-fusiform | 4.07 |
| left-precuneus to right-hippocampus | 3.85 |
| left-middletemporal to left-accumbens-area | 3.83 |
| right-thalamus-proper to right-postcentral | 3.83 |
| left-superiorparietal to left-thalamus-proper | 3.64 |
| left-thalamus-proper to left-putamen | 3.64 |
| left-parstriangularis to left-thalamus-proper | 3.62 |
| left-putamen to left-hippocampus | 3.62 |
| left-lingual to left-superiorparietal | 3.59 |
| right-thalamus-proper to right-lingual | 3.56 |
| right-putamen to right-inferiortemporal | 3.51 |
| left-inferiortemporal to left-putamen | 3.48 |
| left-temporalpole to left-hippocampus | 3.47 |
| right-thalamus-proper to right-hippocampus | 3.45 |
| right-medialorbitofrontal to right-middletemporal | 3.44 |
| left-superiortemporal to left-putamen | 3.38 |
| left-parstriangularis to left-putamen | 3.37 |
| right-putamen to right-superiortemporal | 3.35 |
| left-rostralmiddlefrontal to right-caudalmiddlefrontal | 3.31 |
| right-putamen to right-temporalpole | 3.31 |
| right-hippocampus to right-lingual | 3.28 |
| right-thalamus-proper to right-superiorparietal | 3.25 |
| left-putamen to right-fusiform | 3.24 |
| right-thalamus-proper to right-precentral | 3.24 |
| left-middletemporal to left-putamen | 3.23 |
| right-putamen to right-caudalmiddlefrontal | 3.21 |
| right-caudate to right-postcentral | 3.19 |
| right-putamen to right-superiorparietal | 3.19 |
| right-thalamus-proper to right-parstriangularis | 3.14 |
| right-hippocampus to right-postcentral | 3.14 |
| right-middletemporal to right-supramarginal | 3.14 |
| left-temporalpole to left-Pallidum | 3.09 |
| left-superiorparietal to left-putamen | 3.07 |
| right-putamen to right-postcentral | 3.07 |
| right-putamen to right-parsorbitalis | 3.05 |
| right-fusiform to right-inferiortemporal | 3.03 |
| right-caudate to right-superiorparietal | 3.02 |
| left-isthmuscingulate to left-superiorparietal | 3.01 |
| left-lingual to left-putamen | 3.01 |
| left-caudalmiddlefrontal to right-caudalmiddlefrontal | 3 |
| right-putamen to right-frontalpole | 3 |
| left-precuneus to right-middletemporal | 2.98 |
| **Network using probabilistic MSMT-CSD tracking (p = 0.006)** | |
| left-posterior cingulate to left-superior frontal | 2.99 |
| left-pars opercularis to left-insula | 3.12 |
| left-pars orbitalis to left-thalamus-proper | 3.19 |
| left-pars triangularis to left-thalamus-proper | 4.11 |
| left-postcentral to left-thalamus-proper | 3.02 |
| left-rostral middle frontal to left-thalamus-proper | 3.11 |
| left-superior frontal to left-thalamus-proper | 3.06 |
| left-superior parietal to left-thalamus-proper | 3.06 |
| left-lingual to left-putamen | 3.28 |
| left-middle temporal to left-putamen | 3.28 |
| left-pars orbitalis to left-putamen | 3.03 |
| left-temporal pole to left-putamen | 3.59 |
| left-thalamus-proper to left-putamen | 4.52 |
| left-inferior temporal to left-accumbens-area | 3.67 |
| left-lingual to left-accumbens-area | 3.25 |
| left-middle temporal to left-accumbens-area | 3.42 |
| left-superior temporal to left-accumbens-area | 3.95 |
| left-temporal pole to left-accumbens-area | 3 |
| left-insula to left-accumbens-area | 2.98 |
| left-putamen to left-accumbens-area | 3.87 |
| left-frontal pole to right-caudate | 3.14 |
| left-accumbens-area to right-caudate | 4.13 |
| right-thalamus-proper to right-hippocampus | 3.91 |
| left-caudate to right-accumbens-area | 3.17 |
| right-putamen to right-accumbens-area | 3.33 |
| left-superior frontal to right-caudal middle frontal | 3.13 |
| right-thalamus-proper to right-caudal middle frontal | 3.26 |
| right-thalamus-proper to right-inferior temporal | 3.19 |
| right-putamen to right-inferior temporal | 3.64 |
| right-thalamus-proper to right-lingual | 3.71 |
| left-superior frontal to right-medial orbitofrontal | 3.56 |
| right-inferior parietal to right-medial orbitofrontal | 4.64 |
| left-hippocampus to right-middle temporal | 2.97 |
| right-thalamus-proper to right-middle temporal | 3.69 |
| right-putamen to right-middle temporal | 4.48 |
| right-accumbens-area to right-middle temporal | 3.01 |
| left-postcentral to right-paracentral | 3.38 |
| right-putamen to right-pars orbitalis | 3.31 |
| right-thalamus-proper to right-pars triangularis | 3.13 |
| right-putamen to right-pars triangularis | 3.05 |
| right-thalamus-proper to right-postcentral | 3.9 |
| right-putamen to right-postcentral | 3.36 |
| left-caudal middle frontal to right-posterior cingulate | 3.28 |
| right-thalamus-proper to right-precentral | 3.04 |
| left-posterior cingulate to right-rostral middle frontal | 3.36 |
| left-caudal middle frontal to right-superior frontal | 3.24 |
| left-rostral middle frontal to right-superior frontal | 2.97 |
| right-thalamus-proper to right-superior parietal | 3.07 |
| right-putamen to right-superior parietal | 3.28 |
| right-thalamus-proper to right-superior temporal | 4.01 |
| right-caudate to right-superior temporal | 2.98 |
| right-putamen to right-superior temporal | 4.17 |
| left-superior frontal to right-frontal pole | 3.57 |
| right-thalamus-proper to right-frontal pole | 3.52 |
| right-putamen to right-frontal pole | 3.14 |
| right-putamen to right-temporal pole | 3.31 |
| right-putamen to right-transverse temporal | 3.09 |
| right-pars triangularis to right-insula | 3.07 |

A statistical threshold of *P* = 0.005 (*t* = 2.97, two-tailed *t*-test) was applied to form a set of suprathreshold edges among connected components. Next, the statistical significance of the size of each observed component was evaluated with respect to an empirical null distribution of maximal component sizes obtained under the null hypothesis of random group membership (5000 permutations). *Abbreviations:* CSD, constrained spherical deconvolution; MSMT, multi-shell, multi-tissue; PD, Parkinson’s disease; SSST, single-shell, single-tissue.

**Table S5: Correlation Analysis for the Relationships between Mean Stream Lines of Significantly Changed Subnetworks as Detected by NBS and Clinical Measures (disease duration and UPDRS-III score)**

|  |  | **Disease duration** | | **Motor symptom score** | |
| --- | --- | --- | --- | --- | --- |
|  | **Circuit** | **FDR-corrected p-value** | **r** | **FDR-corrected p-value** | **r** |
| Probabilistic SSST-CSD tracking | motor | 0.99 | 0.05 | 0.21 | −0.41 |
|  | associate | 0.99 | −0.10 | 0.21 | −0.37 |
|  | limbic | 0.99 | 0.07 | 0.41 | −0.21 |
|  | other | 0.99 | 0.03 | 0.41 | −0.19 |
|  | total | 0.99 | 0.01 | 0.21 | −0.34 |
| Probabilistic MSMT-CSD tracking | motor | 0.95 | 0.08 | 0.07 | −0.47 |
|  | associate | 0.95 | 0.02 | 0.07 | −0.44 |
|  | limbic | 0.95 | 0.23 | 0.14 | −0.25 |
|  | other | 0.95 | 0.03 | 0.28 | −0.35 |
|  | total | 0.95 | 0.07 | 0.07 | −0.45 |

*Abbreviations:* CSD, Constrained spherical deconvolution; FDR, False discovery rate; MSMT, multi-shell, multi-tissue; PD, Parkinson’s disease; SSSST, single-shell, single tissue; UPDRS Unified Idiopathic Parkinson’s Disease Rating Scale.
